# Horizontal acquisition of nicotine catabolism gene cluster enhances *Arthrobacter* fitness within tobacco root microbiota

**DOI:** 10.1186/s40168-026-02466-x

**Published:** 2026-07-15

**Authors:** Tomohisa Shimasaki, Yui Nose, Sachiko Masuda, Arisa Shibata, Tsubasa Shoji, Shuhei Yabe, Maiko Furubayashi, Yoshitomo Kikuchi, Ken Shirasu, Kazufumi Yazaki, Yasunori Ichihashi, Akifumi Sugiyama, Ryohei Thomas Nakano

**Affiliations:** 1 Department of Biological Sciences, Faculty of Science, Hokkaido University, Sapporo, Hokkaido 060-0810 Japan; 2 Holobiont and Resilience Research Team, RIKEN Center for Sustainable Resource Science, Tsukuba, Ibaraki 305-0074 Japan; 3 Plant Immunity Research Group, RIKEN Center for Sustainable Resource Science, Yokohama, Kanagawa 230-0045 Japan; 4 Institute of Natural Medicine, University of Toyama, Toyama, Toyama 930-0194 Japan; 5 Biomanufacturing Process Research Center, National Institute of Advanced Industrial Science and Technology (AIST), Hokkaido Center, Sapporo, Hokkaido 062-8517 Japan; 6 Graduate School of Agriculture, Hokkaido University, Sapporo, Hokkaido 060-8589 Japan; 7 Research Institute for Sustainable Humanosphere, Kyoto University, Uji, Kyoto 611-0011 Japan

**Keywords:** Plant specialized metabolites, Tobacco, Nicotine, *Arthrobacter*, Horizontal gene transfer, Bacterial catabolic capacities, Comparative genome analysis, Synthetic microbial communities

## Abstract

**Background:**

Plant roots are hotspots for interactions with soil microbes, where a characteristic bacterial community structure is formed. Plant specialized metabolites often play pivotal roles in this assembly process. However, the molecular basis underlying root microbiota responses to these bioactive compounds, and how such metabolic interactions shape the assembly of host-specific root microbiota, remain largely unknown. Nicotine is a toxic alkaloid predominantly produced by the genus *Nicotiana*, and the genus *Arthrobacter* is known as one of the nicotine-degrading bacteria in the tobacco root microbiota. In this study, we used the tobacco–*Arthrobacter* interaction system as a model and integrated comparative genomics and experimental genetic manipulation assays to uncover the role of bacterial catabolism capacity for host specialized metabolites in shaping host-specific root microbiota.

**Results:**

Nicotine catabolism genes are uniquely found in the *Arthrobacter* strains derived from nicotine-containing environments, and this restricted gene distribution is driven by a plasmid-mediated horizontal gene transfer. To assess the ecological consequences of this genomic adaptation in *Arthrobacter* fitness in tobacco roots, we characterized the nicotine utilization ability of *Arthrobacter* and conducted adaptation assays under *in planta* conditions using genetically manipulated *Arthrobacter* strains and tobacco mutants impaired in nicotine catabolism and biosynthesis, respectively. Nicotine improves *Arthrobacter* colonization of tobacco roots through a catabolism-dependent mechanism. Bacterial community analysis using a synthetic community approach further demonstrated that this metabolic adaptation enhances *Arthrobacter* fitness within tobacco root microbiota.

**Conclusions:**

Our findings illustrated that bacterial catabolic capacity toward host-derived plant specialized metabolites is key for successful root colonization. This metabolic adaptation is driven by plasmid-mediated horizontal gene transfer and ultimately shapes the structure of the root microbiota community.

Video Abstract

**Supplementary Information:**

The online version contains supplementary material available at https://doi.org/10.1186/s40168-026-02466-x.

## Background

Plant roots interact with diverse soil microorganisms, forming a structured community known as the root microbiota. These root-associated microbes significantly affect plant growth and health by producing plant hormones, improving nutrient uptake, and activating host immunity [1, 2]. Root microbiota profiles vary among plant species at lower taxonomic ranks [3–5], although they eventually converge at the higher levels, such as the order [6–8]. A two-step selection model was hypothesized to explain the assembly process of this characteristic bacterial community structure in the roots [9]. In this model, rhizodeposits serve as initial substrates that shift the soil microbiome community characterized by a core plant microbiome, which is then fine-tuned at lower taxonomic levels by host genotype factors. Accordingly, root microbiota harbor characteristic genomic features that enable adaptation to host-specific root environments, and such genetic adaptation may underlie the assembly of plant species-specific root microbiota. Indeed, commensal bacterial strains isolated from *Arabidopsis thaliana* or *Lotus japonicus* roots showed a preferential colonization to their native host rather than non-native host, although the taxonomic compositions of both synthetic communities (SynComs) were identical [5].

Root exudates play fundamental roles in assembling the root microbiota. Plant roots secrete up to 25% of their photosynthates into the rhizosphere as root exudates, which contain primary metabolites (such as sugars, amino acids, and organic acids) as well as plant specialized metabolites (PSMs) [10–13]. These metabolites recruit or repel soil microbes, resulting in the formation of characteristic bacterial communities in the roots. While primary metabolites are generally conserved across the plant kingdom, PSMs are produced in a lineage-specific manner and are therefore assumed to contribute to plant species-specific root microbiota assembly. In *Arabidopsis thaliana,* for instance, biosynthetic mutants impaired in coumarins [14, 15], triterpenes [16], or tryptophan-derived metabolite pathways [17, 18], harbor root microbiota distinct from those of wild-type plants. Similar modulatory effects have been shown for PSMs such as tomatine in tomato (*Solanum lycopersicum*) [19], triterpene in cucumber (*Cucumis sativus*) [20], and benzoxazinoids (BXs) and flavonoids in maize (*Zea mays*) [21, 22]. Although comparisons between wild-type and biosynthetic mutants using culture-independent amplicon sequencing have well described the impact of PSMs on root microbiota communities, the bacterial genetic basis underlying community shifts driven by PSMs secretion in the root microbiota remains largely unknown.

We previously showed that the nicotine-catabolism genes of the genus *Arthrobacter* are associated with the preferential colonization of tobacco (*Nicotiana tabacum*) roots [23]. Nicotine, a toxic alkaloid predominantly found in *Nicotiana* species, contributes to defense against insect herbivores [24, 25]. Although most of the nicotine synthesized in roots is subsequently translocated to the leaves [26, 27], a portion is also secreted into the rhizosphere [28]. We found that the genus *Arthrobacter* is a predominant taxon in tobacco roots and becomes enriched in soils treated with nicotine [23]. This bacterial genus catabolizes nicotine via the nicotine-catabolism (*nic*) gene cluster [29, 30] (Figs. S1a and b). Comparative genome analyses demonstrated a prevalent distribution of the *nic* gene cluster in *Arthrobacter* strains derived from nicotine-containing environments, including tobacco roots and nicotine-treated soils. Recent studies have reported similar metabolic interactions, in which root microbiota catabolizes host-derived PSMs, in soybean (*Glycine max*), tomato, sesame (*Sesamum indicum*), and maize root microbiota [31–33]. These findings suggest that catabolic genes for host-specific PSMs are key to bacterial fitness in their host plant; however, whether these catabolic capacities directly contribute to host interactions and affect overall microbiota structure remains unclear.

Here, we investigated how bacterial catabolic capacities for host-derived PSMs shape both bacterial adaptation to host roots and root microbiota assembly, using the tobacco–*Arthrobacter* interaction system as a model. Comparative analysis of high-quality *Arthrobacter* genomes indicated that horizontal acquisition of the *nic* gene cluster is key for bacterial fitness in tobacco roots. To validate this genomic inference, we employed reverse genetics in both host and bacterial mutants impaired in nicotine biosynthesis and catabolism, respectively. Combined with gnotobiotic inoculation and SynCom experiments, we proposed a mechanistic model explaining the assembly process of plant species-specific root microbiota. This model suggests that the assembly is mediated by the biosynthesis of host PSMs and their subsequent catabolism by the root microbiota.

## Methods

### Bacteria, plant, and chemical materials

The bacterial strains used in this study and their growth media are listed in Dataset S2. Except for NtRootC10, all strains were grown at 28 °C in Luria–Bertani (LB) medium. NtRootC10 was grown at 28 °C in TY medium (5 g/L tryptone, 3 g/L yeast extract) supplemented with 10 mM CaCl_2_. *Escherichia coli* DH5α was grown at 37 °C in LB medium supplemented with appropriate antibiotics (50 μg/mL kanamycin). Seeds of *N. tabacum* cv. Petit Havana line SR-I were provided by Japan Tobacco, Inc. (Tokyo, Japan). Tobacco plants with the *erf189*/*erf199* mutant allele were generated using Petit Havana line SR-I as the genetic background in a previous study [34]. Chemicals were obtained from Wako Pure Chemical Industries (Japan) or Nacalai Tesque (Japan), unless otherwise indicated in Dataset S2.

### Isolation of root- and soil-derived bacteria strains

*Arthrobacter* strains were isolated from the soil (gray lowland soil) collected from a field at the Kyoto University of Advanced Science (KUAS), Kameoka, Kyoto, Japan (34°999380N, 135°559140E) with 9 independent experiments (Isolation1–9; Dataset S4). The detailed methods for Isolation 2–9 can be found in Shimasaki et al., 2021 and Nakayasu et al., 2021, respectively [19, 23]. In this study*,* we further isolated bacterial strains from tobacco roots grown in a growth chamber condition. Tobacco seeds (*Nicotiana tabacum* cv. Burley21) were surface sterilized with 70% ethanol (EtOH) for 1 min and 1% sodium hypochlorite (NaClO) for 10 min and rinsed 5 times with sterile water. Plants were germinated on Murashige and Skoog (MS) medium supplemented with 0.8% agar. Plants were grown in a growth chamber set at 25 °C for 3 days in darkness and 7 days under long-day conditions (16 h light/8 h dark), transferred to plastic pots filled with soil from KUAS fertilized with Hyponex (Hyponex Japan, Japan), and grown for 5 weeks. After washing with tap water, roots were sectioned into smaller fragments and then homogenized with a mortar and pestle in PBS. Homogenates were diluted and plated onto LB agar, and incubated for up to 7 days at 28 °C. Single colonies were picked from plates, sub-cultured on LB agar, and preserved in a 25% glycerol solution at −80 °C. Genomic DNA was extracted by the hot-alkaline DNA extraction method using extraction buffer (25 mM NaOH, 0.2 mM EDTA, 40 mM Tris–HCl [pH 6.8]) [35]. The 16S *rRNA* genes were amplified using primer set 16S_10F and 16S_1500R (Dataset S3). PCR products were purified with the Wizard genomic DNA purification kit (Promega, Madison, USA) according to the manufacturer’s protocol and directly sequenced using primer 10 F for taxonomic identification.

### Whole-genome sequencing and comparative genome analysis

Genomic DNA extraction, whole-genome sequencing, and assembly followed previously described procedures [23]. Comparative genome analysis incorporated publicly available *Arthrobacter* genomes retrieved from the Integrated Microbial Genomes & Microbiomes system (https://img.jgi.doe.gov) and the NCBI (https://www.ncbi.nlm.nih.gov) between April 2021 and April 2022. The prediction and annotation of putative open reading frames (ORFs) of each genome was performed using Prokka [36]. High-resolution phylogenetic inference was conducted using OrthoFinder based on predicted protein sequences [37]. Species tree reconstruction was performed using the STAG [38] and STRIDE [39] algorithms implemented in OrthoFinder. The identification of homologous *nic* genes was performed by BLASTP using amino acid sequences of *Paenarthrobacter nicotinovorans* DSM420 (Dataset S5) against the proteome predicted by the *Arthrobacter* genome sequences (E-value thread > 1 × 10^–15^). Phylogenetic trees and associated data were visualized using ggtree [40]. To infer the host range of plasmid-mediated transfer of the *nic* gene cluster, we performed a phylogenetic analysis of the *nic* gene cluster based on nine conserved *nic* genes (Dataset S5). Protein sequences of each *nic* gene were aligned individually using MAFFT [41], and the resulting alignments were concatenated into a single alignment prior to phylogenetic tree construction with FastTree [42]. The congruence between resulting *nic* gene phylogeny and the species tree inferred by OrthoFinder was assessed using the dendextend package [43].

### Plasmid construction and generation of gene knockout mutants

All constructs and primers used in this study are listed in Dataset S3. All insertion mutants of strain NtRootA2 were generated via homologous recombination. DNA fragments containing partial sequences of target genes were amplified via PCR using the genomic DNA of NtRootA2 as template and cloned into pCR4-TOPO vector (Thermo Fisher Scientific, USA) by TA cloning according to the manufacturer’s protocol. Transformation of NtRootA2 was performed by electroporation as described in Niu et al. [44]. For electrocompetent cell preparation, *Arthrobacter* sp. NtRootA2 was cultured in 100 mL of LB medium at 30 °C until OD_600_ reached 0.3–0.5. Penicillin G was added to the medium at a final concentration of 30 μg/mL, and cultures were incubated for 1 h before harvesting via centrifugation (4 °C, 4000 × g, 10 min). Cells were washed three times with ice-cold electroporation buffer (0.5 M sorbitol containing 10% glycerol), resuspended, and stored at −80 °C. Electrocompetent cells were mixed with 1 μg of plasmid DNA, and pulsed using a MicroPulser electroporator (Bio-Rad, USA) in a 2-mm cuvette at 2.5 kV, 600 Ω, and 10 μF. The bacterial cells were immediately transferred to 900 μL of LB and incubated for 8 h at 30 °C. The insertion mutants were selected on LB medium supplemented with 100 μg/mL kanamycin, and target gene disruptions were confirmed via PCR amplification (Figs. S1c and d).

### Conjugation experiment

To select transconjugants, a spontaneous rifampicin-resistant mutant of *Arthrobacter* sp. StoSoilB3 was generated. StoSoilB3 was grown overnight in LB at 28 °C and subcultured in LB containing increasing concentrations of rifampicin (10, 25, 50, 100, 150, and 200 µg/mL). Stable rifampicin-resistant mutants were selected on LB agar plates containing 200 µg/mL rifampicin. The rifampicin-resistant mutant of StoSoilB3 and the vector control (VC) mutant of NtRootA2 were grown in LB media supplemented with 200 µg/mL rifampicin or 100 µg/mL kanamycin, respectively. After three washes with sterile distilled water, each strain was resuspended in sterile distilled water at OD_600_ = 1.0 and mixed. The bacterial mixture was then spotted onto 0.22-μm membranes (Merck Millipore, United States) placed on LB agar plates and incubated at 28 °C overnight. The filter membranes were transferred to 1.5 mL tubes and washed with 1 mL LB medium by pipetting. To select transconjugants, 20 μL of bacterial suspension was plated on LB medium supplemented with 100 μg/mL of kanamycin and 200 μg/mL rifampicin. Horizontal plasmid transfer was confirmed via PCR and a nicotine degradation assay. Primers were designed to amplify the *ndhL* gene located on the pNtRootA2-2 plasmid and to the genomic regions unique to NtRootA2 and StoSoilB3 (Fig. S2 and Dataset S3).

### Measurements of nicotine-degrading activities using resting cells

Bacterial strains were cultured at 28 °C in their respective media until reaching the stationary phase. Cells were collected by centrifugation at 13,000 × *g* for 2 min and washed twice with PBS buffer (137 mM NaCl, 1.8 mM KH_2_PO_4_, 10 Na_2_HPO_4_•12H_2_O, 2.7 mM KCl, pH 7.4). Pellets were resuspended in 2 mL PBS containing 1 mM nicotine at OD_600_ = 1.0. For SynCom degradation assays, equivalent amounts of each strain (OD_600_ = 1.0) were combined. After incubation for 2 days at 28 °C, supernatants were collected by centrifugation at 13,000 × *g* for 2 min, filtered through a 0.45-μm Minisart RC4 filter (Sartorius, Germany), and stored at −30 °C until ultra-high-performance liquid chromatography (UPLC) analysis.

The microbial reaction products were analyzed using an Acquity UPLC H-Class PLUS system equipped with a photodiode array (PDA) detector (Waters, United States) or a D-2000 Elite UPLC system with an L-2455 photodiode array detector (Hitachi, Japan). For HPLC analyses, 2 μL of each sample was injected onto an Atlantis Premier BEH C18 column (1.7 μm, 2.1 × 100 mm; Waters, United States) with a UPLC BEH C18 VanGuard Precolumn (1.7 μm, 2.1 × 5 mm^2^, Waters, United States). Mobile phases were 10 mM NH_4_HCO_3_ (pH 9.0; phase A) and acetonitrile (phase B). Elution was performed at 0.2 mL/min with 10% B isocratic for 0.25 min, followed by a linear gradient from 10 to 98% B over 6.75 min and 98% B isocratic for 2 min. For analyses on the D-2000 Elite HPLC system, 2 μL of each sample was injected onto a TSKgel ODS-80TM column (5.0 μm, 4.6 × 250 mm^2^; Toso, Japan). Elution was set at 0.2 mL/min with 40% B isocratic for 0.25 min, followed by a linear gradient from 40 to 98% B over 4.0 min and 98% B isocratic for more than 2 min. All analyses were conducted with the column oven temperature at 40 °C, and UV absorbance at 264 nm was monitored using the PDA detector.

### In vitro bacterial growth assay

For growth assays using nicotine as the sole carbon source, the wild type, Δ*ndhL*, and VC mutants of NtRootA2 were grown in LB medium at 28 °C. Cells were harvested via centrifugation at 13,000 × *g* for 1 min, washed with minimal medium (1 × M9 minimal salt supplemented with 0.01% yeast extract, 0.01% MgSO_4_•7H_2_O, 0.005% CaCl_2_•2H_2_O, 0.00033% Fe(Ⅲ)-EDTA), and resuspended in minimal medium containing 5 mM nicotine (CAS: 54-11−5) at OD_600_ = 1.0. After overnight incubation at 28 °C, cells were washed again, adjusted to OD_600_ = 0.1 in minimal medium containing 5 mM nicotine, and cultured at 28 °C. For time-course bacterial growth analysis, 50 μL of culture supernatant was collected at 0, 2, 4, 8, 10, 12, and 16 h, centrifuged at 13,000 × *g* for 1 min, and resuspended in 50 μL of minimal medium. Growth of cultures was monitored based on OD_600_. Minimal medium supplemented with 5 mM glucose served as the control to confirm baseline growth capacity.

For growth assays in rich medium supplemented with nicotine, the wild type, Δ*ndhL*, and VC mutants were cultured in 2 ml TY medium at 28 °C. Overnight cultures were washed with 10 mM MgCl_2_ and adjusted to OD_600_ = 0.001. Then, 2 μL culture was added to 200 μL TY medium containing 1, 10, or 100 μM nicotine in a 96-well microplate. Growth curves were recorded at 600 nm every 15 min at 28 °C for 24 h using a Varioskan LUX plate reader (Thermo Fisher Scientific, USA). For growth assays with other SynCom members and *E. coli* DH5α, growth curves were recorded at 600 nm every 15 min at 30 °C for 30 h using a LogPhase 600 (BioTek, USA). The growth of SynCom members was tested with 100 μM nicotine, and 100 or 1000 μM nicotine was used for *E. coli* DH5α. For statistical analysis, the area under the curve was calculated using the bayestestR package [45].

### Bacterial colonization experiments

Bacterial strains were cultured at 28 °C in LB medium to stationary phase, and then washed and resuspended in 10 mM MgCl_2_ at OD_600_ = 1.0. For competition assays, equivalent amounts of each bacterial strain were combined. Next, 8 μL of this suspension was added to 80 mL of 1/2 × MS medium to obtain a final OD_600_ of 0.0001, then poured into polycarbonate plant boxes (φ87 × ~121.5 mm, Bio Medical Science Inc., Japan) containing approximately 18 g of vermiculite (Fig. S3). Prior to inoculation assay, plant boxes filled with vermiculite were sterilized by the autoclaving at 121 °C for 20 min. Sterilized tobacco seeds were germinated and grown on 1/2 × MS medium with 0.8% agar, and 10-day-old seedlings were transferred to plant boxes and grown for 20 days under long-day conditions (16 h light/8 h dark) at 25 °C.

After 20 days of cultivation, the whole roots were washed with sterile water to remove surrounding vermiculite and placed in a 2 mL tube containing 1 mL 10 mM MgCl_2_ and 5–6 glass beads (2.3 mm). Samples were vortexed for 10 min to release root-colonizing bacteria, after which roots were transferred to a new 1.5 mL tube and weighed. The remaining suspension was diluted 1000-fold and plated on LB agar plates and selection media. After 48 h of incubation at 28 °C, colony-forming units (CFUs) were counted. For competitive colonization assays between wild-type NtRootA2 (Kan^S^) and the Δ*ndhL* or VC mutants (Kan^R^), wild-type CFUs were calculated by subtracting mutant CFUs (counted on LB medium containing 100 μg/mL kanamycin) from the total CFUs (counted on LB medium). For statistical analysis, the log-transformed competitive index (logCI) was calculated as log (CFU of wild-type/CFU of counter mutant).

### SynCom inoculation and bacterial community analysis using 16S *rRNA* gene amplicon sequencing

All bacterial strains were cultured at 28 °C in their respective media to stationary phase, collected by centrifugation at 13,000 × *g* for 2 min and washed twice with 10 mM MgCl_2_. Pellets were resuspended in 10 mM MgCl_2_ to OD_600_ = 1.0, and equal volumes of each strain were combined. Then, 8 μL of suspension was added to 80 mL of 1/2 MS medium (final OD_600_ of 0.0001) and poured into polycarbonate plant boxes (φ87 × ~121.5 mm, Bio Medical Science Inc., Japan) filled with approximately 18 g of sterilized vermiculite. Prior to inoculation assay, plant boxes filled with vermiculite were sterilized by the autoclaving at 121 °C for 20 min. Sterilized tobacco seeds were germinated on 1/2 × MS medium with 0.8% agar, and 10-day-old seedlings were transferred to plant boxes and grown for 20 days under long-day conditions (16 h light/8 h dark) at 25 °C.

Collected tobacco roots were washed with sterile water to remove residual vermiculite, flash-cooled in liquid nitrogen, and ground at 3000 rpm for 15 s using a Multi-Beads Shocker (Yasui Kikai Co., Japan). Fresh root weight for digital PCR analysis was measured after removing residual vermiculite. Total DNA was extracted from the disrupted root tissues using the DNeasy PowerSoil Kit (Qiagen, Germany) according to the manufacturer’s protocol. Libraries were prepared using the two-step PCR amplification protocol of Kumaishi et al. [46]. The QIIME2 platform (version 2021.04) was used for sequence data analysis [47]. For all paired reads, the first 20 bases of both sequences were trimmed (to remove primer sequences), and the bases after 220 were truncated (to remove low-quality sequence data). Potential amplicon sequencing errors were corrected using DADA2 to produce an ASV dataset [48]. Obtained ASVs were aligned using MAFFT [41], and a phylogenetic tree was contracted using FastTree [42]. Each ASV was assigned using a naïve Bayes classifier from the SILVA 138 rRNA database [49], and then the reads that were not assigned to SynCom members were removed; these reads accounted for less than 0.0001% of the total reads. Obtained data was normalized by rarefying to 1000 reads per sample for community analysis. The calculation of UniFrac distances was performed using the QIIME2 platform.

### Statistical analysis for microbiome profiling

All statistical analyses were performed in R software. Constrained principal coordinates analyses were performed based on weighted UniFrac distances using the capscale functions in the VEGAN package [50]. PERMANOVA (999 permutations) based on weighted UniFrac distances was performed to evaluate the effects of plant genotype and bacteria phenotype on community composition (model: beta diversity ~ plant genotype * SynCom phenotype).

### Absolute quantification of NtRootA2 colonization levels by digital PCR

The total copy number of the *socD* gene from strain NtRootA2 in the extracted DNA was quantified using the QIAcuity Digital PCR System (Qiagen, Germany). PCR amplification was performed in a 12 μL reaction mixture containing 1 μL template DNA, 4 μL QIAcuity EvaGreen PCR Master Mix (Qiagen, Germany), 0.6 μL each primers A02_socD_F and A02_socD_R primers (10 μM), and 5.8 μL RNase-free water. PCR conditions were 95 °C for 2 min; 30 cycles of 95 °C for 15 s, 62 °C for 15 s, and 72 °C for 15 s, followed by 40 °C for 5 min. Copy number the *socD* gene per microliter of extract was corrected for elution volume and normalized to root fresh weight.

## Results

### The genus *Arthrobacter* has acquired the *nic* gene cluster via horizontal gene transfer

**Fig. 1:**
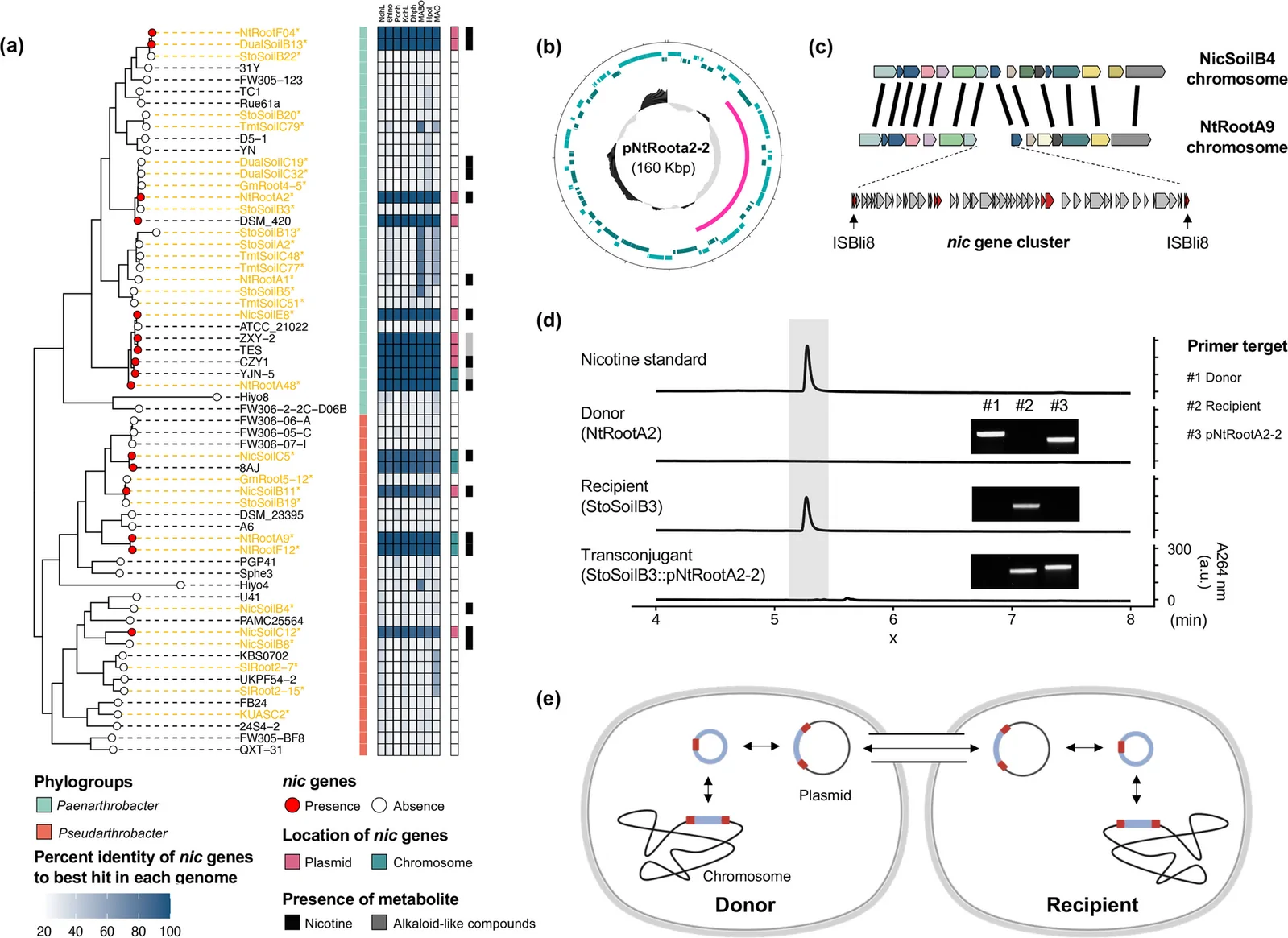
Genomic rearrangements in the genus *Arthrobacter* via horizontal transfer of the *nic* gene cluster. **a** Species tree of the genus *Arthrobacter* showing the phylogenetic distribution of the *nic* gene cluster. The phylogenetic tree of 62 *Arthrobacter* genomes was inferred by Orthofinder. *Arthrobacter* strains isolated in this study are marked in orange with an asterisk. Leaf nodes indicate presence or absence of the *nic* gene cluster. The inner bar represents the latest classification of the genus *Arthrobacter*. The heatmap shows percent identity of best BLASTP hits in each genome to the *nic* genes from *Paenarthrobacter nicotinovorans* pAO1 [53]. The inner bar adjacent to the heatmap on the right indicates the location of the *nic* gene cluster. The outer bar shows the presence of nicotine or pesticides in the isolation environment. **b** Diagram of the *nic* gene cluster of strain NtRootA2 located on pNtRootA2-2 plasmid. From inside to outside, the inner magenta bar indicates the genomic region encoding the *nic* gene cluster, followed by GC content, forward-strand coding DNA sequences (CDSs), reverse-strand CDSs. **c** Diagram of the composite transposon carrying the *nic* gene cluster in strain NtRootA9. IS elements within the *nic* gene cluster are colored red. The *nic* gene cluster is flanked by IS elements ISBli8 (black arrows), forming a composite transposon. The flanking genomic regions of the *nic* gene cluster are shown with block arrows. The homologous genomic regions of non-nicotine-degrading strain NicSoilB4 were shown in the same color and connected by black lines. **d** Nicotine degradation assay and PCR amplifications of the donor, recipient, and transconjugant. Culture supernatants from strains grown in the nicotine-supplemented PBS buffer were analyzed via UPLC. All chromatograms were recorded at 264 nm using identical intensity scales. a.c., arbitrary units. Primer pairs amplified specific genomic regions of NtRootA2 and StoSoilB3, as well as the *ndhL* gene on pNtRootA2-2 plasmid. **e** Proposed model of genomic rearrangements in the genus *Arthrobacter* mediated by horizontal transfer of the *nic* gene cluster. The *nic* gene cluster can transfer to different genomic regions via a composite transposon within a single cell, and once integrated into conjugative plasmids, can transfer to other bacterial cells

We previously demonstrated that the distribution of the *nic* gene cluster within the genus *Arthrobacter* is strongly associated with the presence of nicotine or related metabolites in their source environments [23]. To gain deeper insight into the ecological functions and evolutionary trajectory of this gene cluster, we isolated *Arthrobacter* strains from the roots of tobacco, soybean, and tomato and subjected them to whole-genome sequencing. We also isolated *Arthrobacter* strains from soils treated with different PSMs, including nicotine and santhopine from tobacco [23], and tomatine from tomato [19]. In total, 34 complete *Arthrobacter* genomes were obtained. By integrating publicly available sequences, we established an *Arthrobacter* pan-genome comprising 62 high-quality genomes (Dataset S1). A phylogenetic inference using Orthofinder revealed that the genus *Arthrobacter* is largely divided into two distinct phylogroups, which are consistent with the recently reclassified genera *Paenarthrobacter* and *Pseudarthrobacter*, respectively (Fig. 1a) [51]. Although our *Arthrobacter* strains were isolated from the same soil, the *nic* gene cluster was specifically identified in the strains from nicotine-containing environments, such as tobacco roots or nicotine-treated soils (Fig. 1a). Within the entire *Arthrobacter* pan-genome, the *nic* gene cluster showed a sporadic distribution and was predominantly found in isolates derived from nicotine or alkaloid-like compounds-containing environments, such as pesticides or fungicides (Fig. 1a and Dataset S1; *P* = 2.97 × 10^−6^, Fisher’s exact test). Because several *Arthrobacter* strains carried the *nic* gene cluster on their plasmid (Fig. 1a and b), we inferred that these *Arthrobacter* strains may have acquired it via plasmid-mediated horizontal gene transfer (HGT) [52].

To test whether *Arthrobacter* can acquire the *nic* gene cluster via plasmid transfer, we performed a conjugation assay using a strain NtRootA2, which harbors the *nic* gene cluster on the pNtRootA2-2 plasmid (Fig. 1b). To trace their plasmid transfer, we inserted a kanamycin (Km)-resistance gene into a non-coding region of the pNtRootA2-2 plasmid, positioned outside the *nic* gene cluster (Figs. S1b and d). This modified donor strain was then co-cultivated with a recipient strain (StoSoilB3), which does not possess the *nic* gene cluster, and transconjugants were selected by antibiotic selection (Fig. S2). Subsequent analysis confirmed the presence of pNtRootA2-2 in the transconjugant and demonstrated that it had gained nicotine degradation ability (Fig. 1d).

In contrast, some *Arthrobacter* strains harbor the *nic* gene cluster on their chromosome (Fig. 1a). Notably, *nic* gene clusters in strains NtRootA9 and F12 were flanked by insertion sequences (ISs), and the adjacent genomic region was strikingly conserved in the non-nicotine-degrading strain NicSoilB4 (Fig. 1c and Fig. S4a). This arrangement—DNA located between two IS copies—constitutes a composite transposon, typically associated with intracellular DNA mobility [54]. In fact, GC content in genomic regions flanked by IS elements was lower than that in the surrounding regions in strain NtRootA9 (Fig. S4b). Similar composite transposon-like structures were found on plasmid pZXY21 in strain ZXY-2 and plasmid pTES1 in strain TES (Fig. S4a), implying that mobilization of the *nic* gene cluster by composite transposons is prevalent within the genus *Arthrobacter*.

We therefore propose a transfer model in which the *nic* gene cluster moves between chromosomal and plasmid locations within a cell via composite transposon and is then disseminated among bacterial cells when integrated into conjugative plasmids (Fig. 1e). To assess the host range of HGT, we built phylogenies of *nic* gene cluster based on concatenated alignment sequences of nine conserved *nic* genes (Fig. S5a) and compared them to the species tree (Fig. 1a). While several incongruences were found within the genus *Pseudarthrobacter*, the phylogeny of the *nic* gene cluster was largely congruent with the species tree (Fig. S5b). Notably, even though strains NtRootA9 and NtRootF12 belong to the genus *Pseudarthrobacter*, the protein sequences of their *nic* gene cluster are phylogenetically closer to those of the *Paearthrobacter* group. These results suggest that the host range of the *nic* gene cluster transfer is mostly restricted in closely related species, but it may also be transferred across genera. Together, our findings suggest that HGT of the *nic* gene cluster mediated by mobile genetic elements facilitates *Arthrobacter* adaptation to nicotine-containing environments, including tobacco roots.

### Nicotine affects *Arthrobacter* growth in a catabolism-dependent manner

**Fig. 2:**
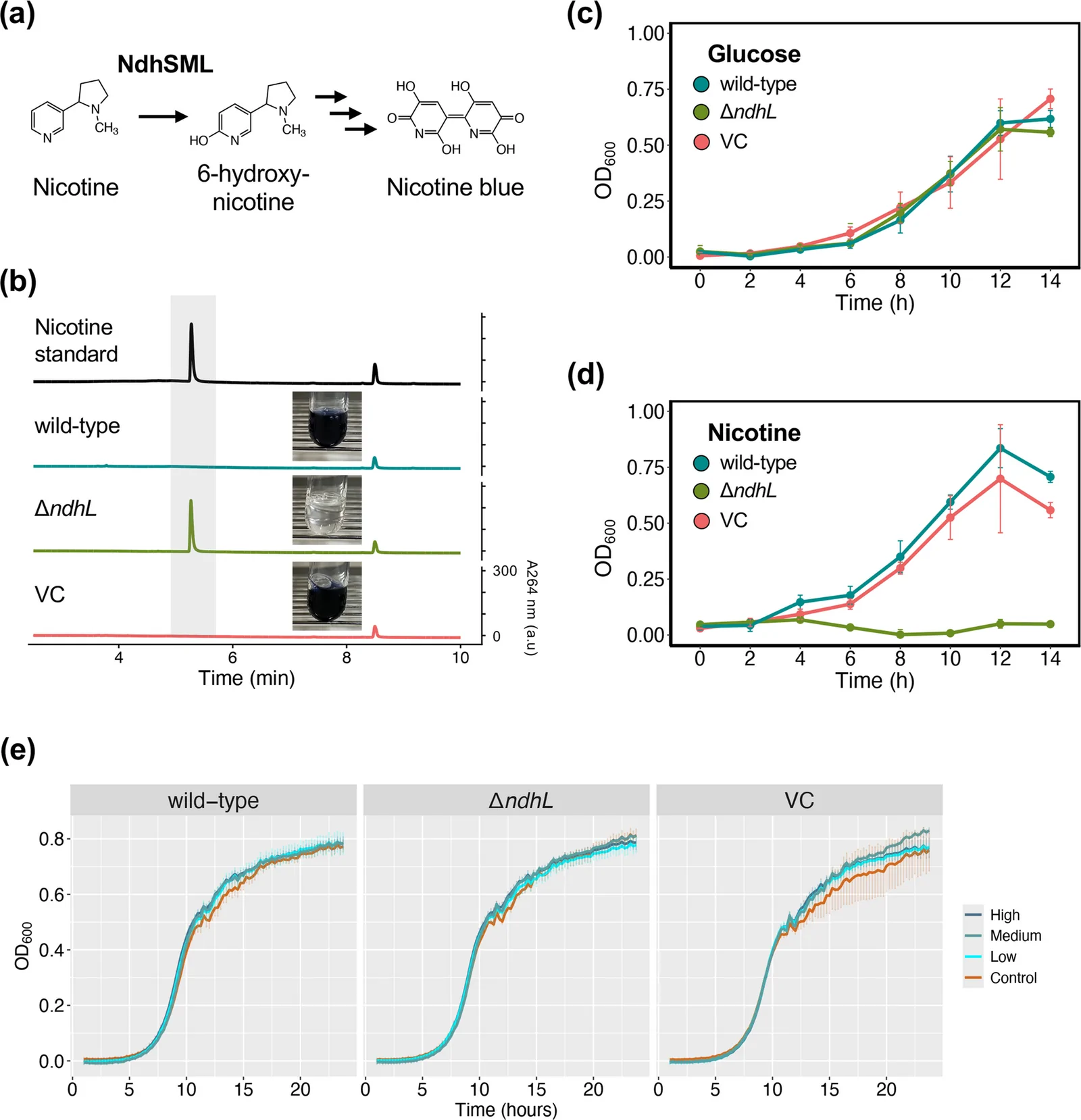
Nicotine affects *Arthrobacter* growth in a catabolism-dependent manner.** a** Partial nicotine degradation pathway in *Arthrobacter*. NdhSML hydroxylates the C6 position of pyridine ring in nicotine to produce 6-hydroxynicotine. Blue-colored intermediates, known as nicotine blue, accumulate during degradation. **b** Nicotine degradation assay of NtRootA2 and its derivative mutants. UPLC analysis of culture supernatants grown in the nicotine-supplemented PBS is shown. All chromatograms were recorded at 264 nm using identical intensity scales. a.c., arbitrary units. Medium of wild-type and VC strains turned blue due to nicotine blue accumulation during nicotine catabolism. Growth rates of the wild-type, Δ*ndhL*, and VC strains of NtRootA2 in the minimal medium supplemented with **c** glucose or **d** nicotine are shown. Error bars indicate Mean ± SD of three replicates. **e** Growth curves (OD_600_) of each *Arthrobacter* isolate in TY medium supplemented with different nicotine concentrations**.** Data are expressed as mean ± SD (*n* = 5). Control: 0 μM nicotine; Low, 1 μM nicotine; Medium, 10 μM of nicotine; and High, 100 μM nicotine. Essentially identical results were obtained from two independent experiments

To assess how the horizontal acquisition of the *nic* gene cluster contributes to *Arthrobacter* fitness in nicotine-containing environments, we constructed an insertional mutant of strain NtRootA2 by integrating vector pCR4-TOPO into the coding region of *ndhL* (Figs. S1b and c), which encodes the enzyme subunit catalyzing the first step of nicotine degradation (Fig. 2a and Fig. S1a). To control for vector insertion effects, we also used the kanamycin-resistant NtRootA2 line produced during the conjugation assay as a vector control in subsequent experiments (Fig. S1d). Nicotine degradation assays confirmed the loss of nicotine degradation ability in Δ*ndhL*, while the VC retained its catabolic capacity (Fig. 2b). Consistently, nicotine blue, an intermediate of nicotine degradation (Fig. 2a), was absent in the Δ*ndhL* mutant, verifying that disruption of *ndhL* impaired nicotine catabolism.

We cultured these strains in minimal medium supplemented with either glucose or nicotine as the sole carbon source. All strains grew to a similar level in glucose, but only Δ*ndhL* failed to grow in nicotine medium (Figs. 2c and d), indicating that NtRootA2 utilizes nicotine as a carbon source via the *nic* gene cluster. Beyond nutrient utilization, recent studies demonstrated that PSMs inhibit bacterial growth, while bacterial catabolism of these compounds increases bacterial tolerance by detoxifying them [33]. We therefore evaluated in vitro bacterial growth of NtRootA2 in nutrient media containing three different nicotine concentrations. Unexpectedly, the growth of neither wild-type nor Δ*ndhL* was affected even in the presence of 100 μM of nicotine (Fig. 2e and Fig. S6), indicating that nicotine primarily affects *Arthrobacter* growth by serving as a nutrient source.

### Nicotine catabolism enhances competitive fitness of *Arthrobacter* in tobacco roots

**Fig. 3:**
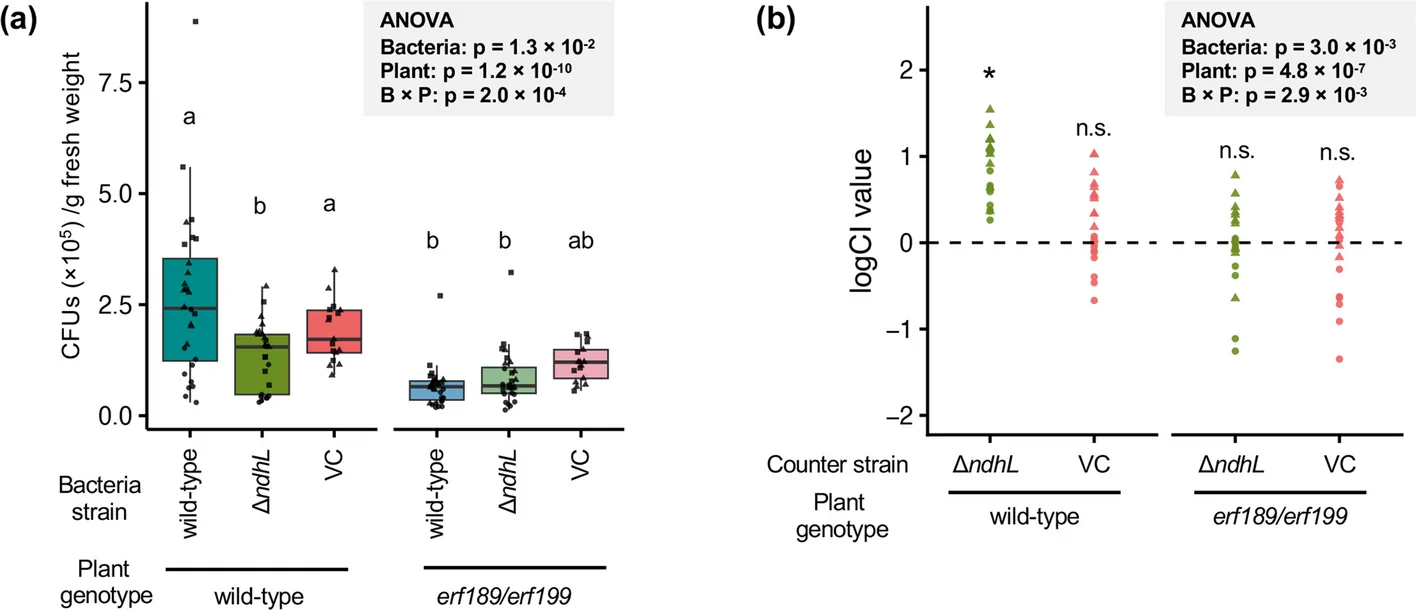
Nicotine-catabolic capacity contributes to *Arthrobacter* fitness in the tobacco roots.** a** Colonization assay of NtRootA2 and its derivative mutants in a mono-association system. These *Arthrobacter* strains were inoculated into the wild-type or *erf189*/*erf199* tobacco roots. Data are from three independent experiments, except for two groups (VC in the wild-type roots and VC in the *erf189*/*erf199* roots), which were performed twice (*n* = 17–30). Different letters indicate statistical differences corresponding to two-way ANOVA with Tukey’s honest significant difference (HSD) test (*α* = 0.05). Different shapes depict data points from full-factorial biological replicates. Bacteria: Bacteria strain (wild-type, Δ*ndhL*, or VC). Plant: tobacco genotype (Wild-type or *erf189*/*erf199*). “B × P”: interaction between bacteria phenotype and tobacco genotype. **b** Root colonization competition assay between the wild-type and Δ*ndhL* or VC strains in the roots of wild-type or *erf189*/*erf199* mutant tobacco. Data are from two independent experiments (*n* = 20–22). Asterisks indicate statistical significance determined by two-way ANOVA on log-transformed competition indices (logCI), followed by model-based tests using estimated marginal means (emmeans) to evaluate deviations from neutral competition (logCI = 0), with Holm-adjusted *P*-values for multiple comparisons (*P* < 0.05). n.s., not significant

In vitro growth assays showed that nicotine affects *Arthrobacter* growth in a catabolism-dependent manner. Next, we examined root colonization by the wild-type and mutant strains to determine the impact of nicotine-catabolic capacity on *Arthrobacter* adaptation to tobacco roots. In order to clarify the contributions of host-derived nicotine, we also employed a double-knockout mutant of jasmonate (JA)-responsive ETHYLENE RESPONSE FACTOR (ERF) transcription factor genes *ERF199* and *ERF189* (*erf189*/*erf199*) [34], which produces significantly less amount of nicotine in leaves and roots. In wild-type tobacco roots, Δ*ndhL* colonized at significantly lower levels than the wild type and VC (Fig. 3a). However, in the *erf189*/*erf199* roots, all strains colonized at comparable levels. Two-way analysis of variance (ANOVA) demonstrated significant effects of both *Arthrobacter* catabolism and plant nicotine biosynthesis on *Arthrobacter* colonization, with a significant interaction. Notably, host nicotine biosynthesis had a greater influence on *Arthrobacter* colonization than bacterial catabolism (*R*^*2*^ Plant = 0.26, *R*^*2*^ Bacteria = 0.06, two-way ANOVA). We then investigated the role of nicotine catabolism under competitive conditions within the root niche by co-inoculating the wild-type and either Δ*ndhL* or VC strains into wild-type or *erf189*/*erf199* mutant tobacco roots. In the wild-type tobacco roots, Δ*ndhL* showed significantly reduced colonization relative to the wild-type, whereas the VC colonized at a comparable level (Fig. 3b). No competitive differences were observed in both pairs of strains inoculated into the *erf189*/*erf199* roots. Together, these results indicate that nicotine catabolism enhances *Arthrobacter* fitness in tobacco roots, particularly under competitive conditions.

### Metabolic adaptation to nicotine enhances *Arthrobacter* fitness and influences the tobacco root microbiota community structure

**Fig. 4:**
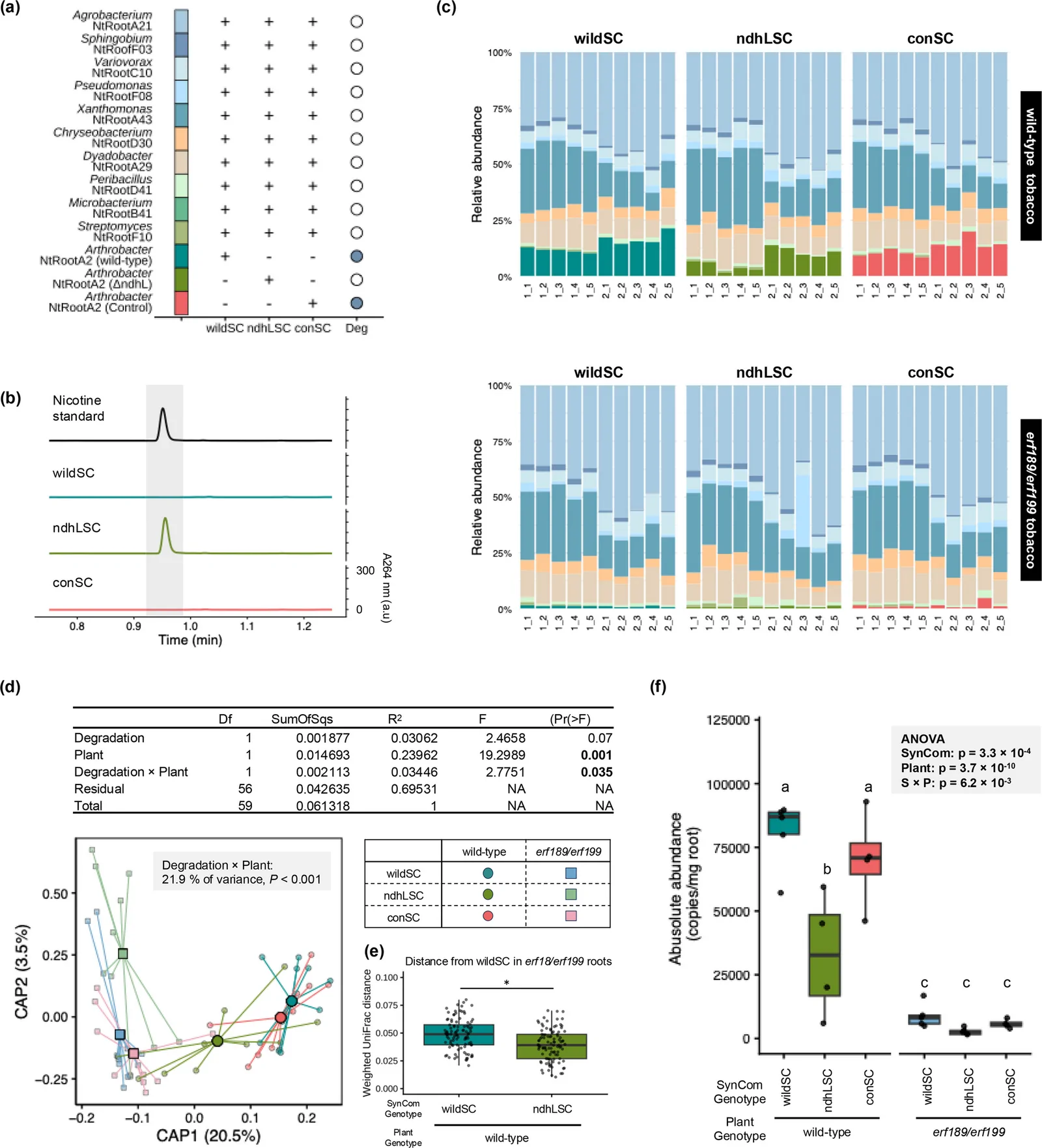
Metabolic adaptation to nicotine enhances* Arthrobacter* fitness and influences the tobacco root microbiota community structure.** a** Bacterial strains used in the synthetic community (SynCom). Strains included in each SynCom are marked “ + ” and excluded strains “ − ”. Detailed taxonomic information is shown in Dataset S2. The right column indicates the nicotine degradation ability of each bacterial strain. Deg, nicotine degradation. **b** Nicotine degradation assay of each SynCom. HPLC analysis of culture supernatants from SynCom grown in nicotine-supplemented PBS is shown. All chromatograms were recorded at 264 nm using identical intensity scales. a.c., arbitrary units. Essentially identical results were obtained from two independent experiments. **c** Bacterial community composition of each SynCom inoculated into the wild-type or *erf189*/*erf199* tobacco roots. **d** Constrained principal coordinates analysis of the community structure of SymComs in wild-type and *erf189*/*erf199* roots based on weighted Unifrac distance. Data are from two independent experiments. Ordination was constrained by the model ~ Degradation phenotype × tobacco genotype. Degradation: SynCom phenotype (“Degrading SynCom” [wildSC, conSC] or “Non-degrading SynCom” [ndhLSC]). Plant: tobacco genotype (Wild-type or *erf189*/*erf199*). Degradation × Plant: interaction between SynCom phenotype and tobacco genotype. PERMANOVA results based on weighted UniFrac distance for variation in bacterial community structure are shown in the upper table. **e** Comparison of weighted UniFrac distance. Box plots showing mean and variance of average pairwise weighted UniFrac distances between samples of wildSC inoculated into *erf189*/*erf199* tobacco roots and samples of wildSC- or ndhLSC- inoculated into wild-type tobacco roots (*n* = 100). Asterisks indicated statistical significance (*t*-test, *P* < 0.05). **f** Absolute abundance of strain NtRootA2. Individual data points are indicated with dots (*n* = 4–5). The result table for two-way ANOVA is included. SynCom: wildSC, ndhLSC, or conSC. Plant: tobacco genotype (Wild-type or *erf189*/*erf199*). S × P: interaction between SynCom and tobacco genotype. Different letters indicate statistical differences corresponding to two-way ANOVA followed with HSD test (*α* = 0.05)

Because *Arthrobacter* catabolism and tobacco biosynthesis of nicotine significantly affected *Arthrobacter* colonization of the tobacco roots, we next investigated whether these metabolic interactions also affect tobacco root microbiota assembly. To address this, we established SynComs comprising 11 phylogenetically diverse bacterial strains isolated from tobacco roots, including the wild-type strain of *Arthrobacter* sp. NtRootA2 (hereafter the wildSC) (Fig. 4a). Two additional SynComs were generated by substituting this wild-type strain with either the Δ*ndhL* or VC strains (ndhLSC and conSC, respectively). Except for the NtRootA2 wild-type and VC strains, none of the SynCom members degraded nicotine (Fig. S7), whereby nicotine-catabolism abilities of *Arthrobacter* strains represented the entire nicotine-catabolism capacity of each SynCom (Fig. 4b). Based on this, the SynComs were classified as “Degrading SynComs” (wildSC and conSC) or “Non-degrading SynCom” (ndhLSC).

These SynComs were inoculated into wild-type or *erf189*/*erf199* tobacco roots and community structures were assessed via 16S *rRNA* gene amplicon sequencing. All strains except *Paenibacillus* NtRootD41 were detected in roots at 20 days after inoculation. *Agrobacterium* NtRootA21 and *Xanthomonas* NtRootA43 were the most dominant, followed by *Dyadobacter* NtRootA29, across both plant genotypes (Fig. 4c and Fig. S8). Permutational analysis of variance (PERMANOVA) revealed significant shifts in overall bacterial community composition driven by plant genotype (*R*^*2*^ = 0.24, *P* = 0.001), whereas SynCom phenotype showed no major effect (*R*^*2*^ = 0.03, *P* = 0.07) (Fig. 4d). Interestingly, despite minor effect of SynCom phenotype alone on the bacterial community structure, we detected a significant interaction between SynCom phenotype and plant genotype (*R*^*2*^ = 0.03, *P* = 0.035, PERMANOVA). Notably, the disruption of *ndhL* shifted community composition toward that formed in the *erf189*/*erf199* roots inoculated with wildSC or conSC (Fig. 4e), which was largely explained by the first axis of the constrained ordination analysis (Fig. 4d). A slight community shift was also detected in the ndhLSC, even in *erf189*/*erf199* roots. This likely reflects the incomplete loss of nicotine biosynthesis in *erf189*/*erf199*, which still accumulates detectable nicotine levels in the roots [34].

The relative abundance of NtRootA2 was significantly reduced in the ndhLSC in wild-type roots and in all SynComs inoculated into the *erf189*/*erf199* roots. Absolute quantification using digital PCR targeting *socD*, a single-copy gene unique to NtRootA2 showed a similar colonization pattern, confirming that these changes are quantitative (Fig. 4f). As in mono-association colonization assays (Fig. 3a), plant genotype exerted a stronger effect on NtRootA2 absolute abundance than SynCom phenotype (*R*^*2*^ Plant = 0.67, *R*^*2*^ SynCom Phenotype = 0.12, two-way ANOVA), with a significant interaction between those factors. On the other hand, both SynCom phenotype and plant genotype had an only mild influence on the populations of other SynCom members (Fig. 4c). Although the relative abundance of *Variovorax* NtRootC10 and *Agrobacterium* NtRootA21 was significantly higher in the *erf189*/*erf199* root (*P* = 0.0325 and *P* = 0.0189, respectively; two-way ANOVA) (Fig. S8), their populations were rarely influenced by the SynCom phenotype. We also observed an interaction between plant genotype and SynCom phenotype on the relative abundance of *Dyadobacter* NtRootA29 (*P* = 0.0292; two-way ANOVA), but no significant differences were detected among the experimental treatments.

**Fig. 5:**
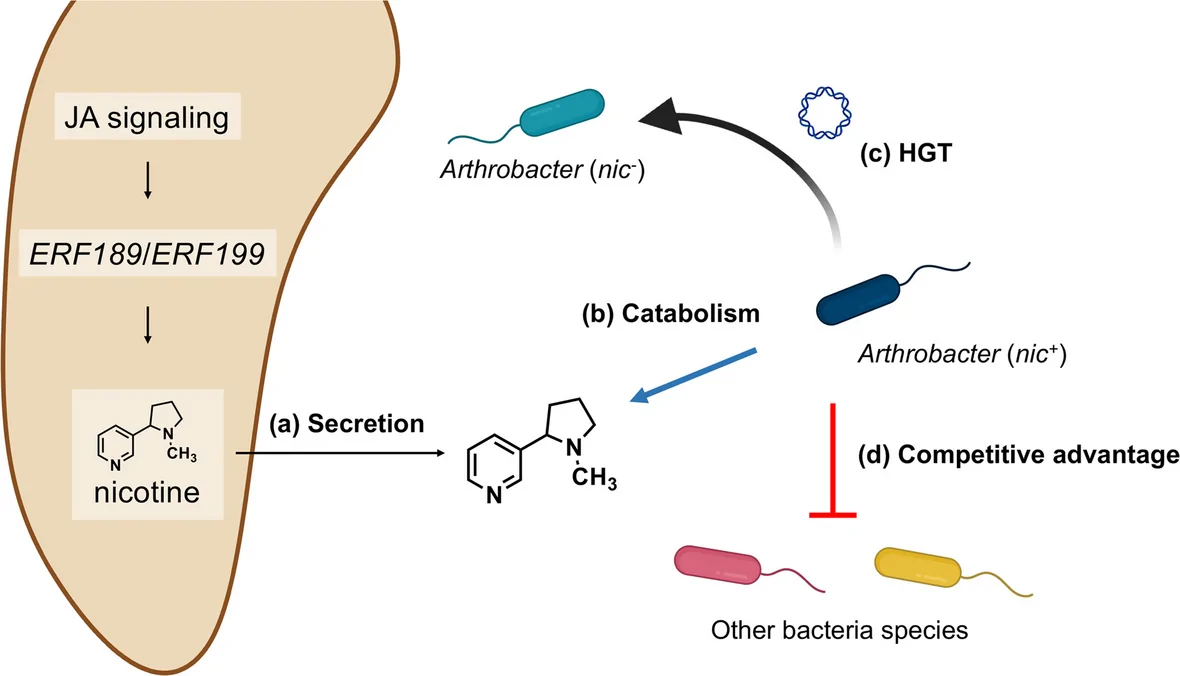
Proposed model of nicotine-mediated assembly of the tobacco root microbiota. Anticipated events related to the interaction between tobacco and its associated microbiota are represented by letters. **a** Nicotine secretion promotes the formation of tobacco-specific root microbiota characterized by the enrichment of *Arthrobacter*. **b** The *nic* gene cluster enables this bacterial adaptation to tobacco roots and **c** this gene cluster can horizontally transfer among the genus *Arthrobacter* via plasmid transfer. Nicotine exerts only a minor influence on the growth of other bacteria. **d** Thereby, nicotine-catabolic capacity confers a competitive advantage on *Arthrobacter* over other microbiota members, shaping a characteristic tobacco root microbiota. The figure was created with Biorender.com

These results suggest that the effect of nicotine on the tobacco root microbiota is largely restricted to the population of *Arthrobacter* and rarely affects those of other bacterial taxa. To assess this point, we monitored the growth of other SynCom members in the presence of 100 μM nicotine. This analysis further demonstrated a minor effect of nicotine on the bacterial growth of SynCom members (Fig. S9). Importantly, it has been shown that native root bacteria tend to exhibit a greater tolerance to the PSMs produced by their host plants [55]. However, we did not observe any growth inhibition activity against *E. coli* DH5α, which was used as a non-native model bacterium, even in the presence of 1000 μM nicotine, suggesting that nicotine is less toxic than previously anticipated to a wide range of bacterial taxa. Collectively, these results indicated that nicotine serves as a selective nutrient source for *Arthrobacter*. This metabolic adaptation confers a competitive advantage on *Arthrobacter* within the root microbiota, underpinning the assembly of characteristic tobacco root microbiota enriched in *Arthrobacter* (Fig. 5).

## Discussions

### The function of PSMs in the assembly of plant species-specific root microbiota

Root exudates contain numerous primary and specialized metabolites that strongly influence root microbiota composition. Here, we provide experimental evidence that PSMs shape the root microbiota by serving as nutrient sources for a specific root microbiota member, thereby conferring a competitive advantage (Fig. 4). Catabolism of host-derived PSMs by root microbiota has also been reported in various plant–bacterial systems [23, 31, 33], indicating that the ability to metabolize PSMs is a common adaptation strategy. Likewise, it is widely appreciated that primary metabolites play a fundamental role in microbial recruitment, as root-associated bacteria generally catabolized amino acids, organic acids, and sugars, more efficiently than non-adapted isolates [56–58]. Large-scale comparative genome studies further revealed that plant-associated bacteria harbor more genes related to carbohydrate metabolism functions compared to non-plant-associated bacteria [59, 60]. Importantly, these primary-metabolic traits are broadly conserved across core root microbiota members. In contrast, PSM catabolic genes are generally restricted to a narrow subset of bacterial taxa [29, 33, 61], and exogenous application of PSMs to soil often enriches those capable of degrading them [62]. Together with our findings, these observations support the two-step selection process whereby PSMs act as host genotype factors that fine-tune root microbiota composition by selectively enriching bacterial taxa with the capacity to metabolize them.

### MGEs play a role in metabolic adaptation of root microbiota

Although we isolated *Arthrobacter* strains from the same soil, the *nic* gene cluster was identified only in strains from tobacco roots or nicotine-treated soils (Fig. 1a). Similarly, the isoflavone catabolism (*ifc*) and benzoxazinoid degradation (*bxd*) gene clusters are confined to root microbiota of legumes and maize, respectively, which produce these PSMs [33, 61]. These findings suggest that PSM catabolic genes are selectively distributed, even among strains within the same bacterial taxa. Our comparative genome study revealed that some *Arthrobacter* strains harbor the *nic* gene cluster on plasmids, and HGT of these plasmids could explain this restricted genomic adaptation (Fig. 1a). In contrast, recently identified PSM catabolic genes such as *ifc* and *bxd* genes exclusively reside on the chromosome. This inconsistency may be explained by the larger size of the *nic* gene cluster, which comprises over 40 genes spanning approximately 50 kb, compared with the smaller *ifc* (~20 genes) and *bxd* (~15 genes) clusters. Since plasmid carriage imposes replication and segregation costs that can reduce bacteria fitness [63, 64], they are selectively maintained under a specific environment where they can confer strong benefits to the host bacteria. It is conceivable that a trade-off between the plasmid carriage cost and the metabolic advantage conferred by the *nic* gene cluster likely explains its restricted phylogenetic distribution within *Arthrobacter*. Interestingly, nicotine degradation pathways largely identical to that of *Arthrobacter* were found in other bacterial genera belonging to the order Actinomycete, including the genera *Rhodococcus* and *Nocardioides* [29]. Previous studies suggested that their *nic* gene clusters originated from a common ancestor and were disseminated across the genera through HGT across the genera [65, 66]. Based on the relatively low amino acid identities among the *nic* gene clusters, it has been inferred that their HGT-mediated divergence may have occurred in the distant past.

Transfers of MGEs have also been reported in other commensal bacteria, and these genomic rearrangement events appear to be facilitated by metabolites present in root exudates [67–69]. For instance, fabricated ecosystems showed conjugative plasmid transfer among *Pseudomonas putida* in the *Brachypodium distachyon* rhizosphere [70], with frequencies increasing in response to host-derived metabolites. Moreover, a long-read metagenomic analysis also uncovered numerous previously unknown plasmids in the rice leaf microbiota [71], highlighting the widespread but underexplored functions of MGEs in the plant–microbiota interactions. Therefore, given our results showing strong effects of HGT on *Arthrobacter* fitness and on tobacco root microbiota composition, functional characterizations of MGEs will further elucidate molecular mechanisms of bacterial host adaptation and root microbiota assembly.

### Potential role of nicotine in shaping the tobacco root microbiota beyond nutrient utilization

Despite the significant contribution of bacterial catabolism of host-derived PSMs in adaptation, the host nicotine-biosynthesis mutant exerted a stronger impact on bacterial colonization than the nicotine-catabolism mutant of *Arthrobacter* (Fig. 4d). In addition to serving as a selective nutrient source, the antibacterial activity of PSMs is a key factor influencing bacterial colonization [55]. However, we did not observe any growth inhibition of our SynCom members at 100 μM nicotine (Fig. S9). Even though nicotine accumulation levels in the tobacco rhizosphere remain to be elucidated, the community shift induced by host genotype is unlikely driven by a toxicity of nicotine, as many other classes of PSMs exert antibacterial activity at around 100 μM [33, 55, 72].

Given the minor effect of nicotine on the other SynCom members (Fig. S9), one possible explanation for this inconsistency is the pleiotropic effects of genetic manipulation of *ERF189/ERF199* genes on tobacco metabolism. Indeed, metabolome analysis revealed increased accumulation of antibacterial compounds, such as chlorogenic acid [73, 74], in the *erf189/erf199* mutants [34]. Alternatively, considering that the impact of host genotype is primarily limited to *Arthrobacter* (Fig. S8), another possibility is that nicotine enhances *Arthrobacter* fitness via uncharacterized mechanisms in addition to its catabolism. Consistent with this idea, it has been demonstrated that nicotine enhances *Pseudomonas aeruginosa* NXHG29 colonization of tobacco roots by acting as a chemoattractant and growth stimulant [28]. Further studies are needed to clarify these points, for example, by employing an additional nicotine-deficient mutant targeting biosynthesis genes or performing a complementation experiment via direct application of exogenous nicotine to the soil.

Beyond adaptation to their host roots, degradation of host-derived PSMs also plays a role in bacteria–bacteria interactions. For example, degradation of isothiocyanates, a characteristic class of PSMs found in *Brassicaceae* plants, influences the growth of non-degrading commensals through cross-feeding of metabolic intermediates or detoxification [72, 75]. It has also been demonstrated that the presence of other bacterial strains can redirect the BX catabolic pathway of BXs of a degrading strain, likely by metabolizing intermediate compounds [76]. Nicotine is similarly metabolized by various bacterial species via at least three distinct pathways [29], producing metabolic intermediates such as nicotine blue and methylamine. Therefore, it is crucial to comprehensively understand how nicotine and its degradation intermediates are metabolized by the whole bacterial community and how these metabolic interactions shape root microbiota.

### Ecological relevance of catabolism-mediated root microbiota assembly

A major remaining question concerns the ecological relevance of nicotine catabolism-mediated assembly of the tobacco root microbiota. It has been well documented that biotic and abiotic stresses often stimulate PSM biosynthesis and secretion [77–79], suggesting that plants may secrete PSMs to establish the beneficial root microbiota that can alleviate these stresses [21, 80, 81]. This PSM-triggered functioning of root microbiota is referred to as a “cry for help” or “microbiome feedbacks” [82, 83]. In this study, we propose that bacterial catabolic capacities for host-derived PSMs contribute to these processes. For instance, cucurbitacins, the characteristic bitter triterpenoids in cucurbit plants, recruit *Enterobacter* and *Bacillus*, thereby enhancing resistance to soil-borne wilt fungal pathogen [20]. Notably, this feedback effect of the *Enterobacter* was mediated by cucurbitacin degradation. *Arthrobacter* colonization can promote plant growth and modulate plant hormone signaling pathways, including auxin and JA signaling [84–86]. Hence, it is intriguing to investigate whether the nicotine catabolism-driven community shift in tobacco roots participates in a “microbiome feedbacks” response to environmental stresses, such as herbivore attacks, which trigger nicotine biosynthesis [25].

## Conclusion

Root microbiota can catabolize various compounds secreted by plant roots, and these catabolic capacities mediate complex plant–microbiota interactions [87]. In this study, we show that the nicotine-catabolic capacity of *Arthrobacter* substantially contributes to its colonization of tobacco roots. Comparative genome analysis demonstrates that MGEs underlie this metabolic interaction between tobacco and *Arthrobacter*. These results support a mechanistic model, in which horizontal acquisition of the *nic* gene cluster improves *Arthrobacter* fitness and confers a competitive advantage over other microbiota members, thereby shaping the tobacco root microbiota characterized by the enrichment of *Arthrobacter* (Fig. 5). This study advances our molecular understanding of how PSMs shape plant-species-specific root microbiota.

## Supplementary Information

Supplementary Material 1: Fig. S1. Nicotine-catabolism pathway and *nic* gene cluster of *Arthrobacter* spp., and generation of NtRootA2 mutants. (a) Overview of the nicotine degradation by *Arthrobacter* spp. and (b) *nic* gene cluster of NtRootA2. The cluster comprises three sub-gene clusters involved in γ-N-methylaminobutyrate catabolism, nicotine degradation, and molybdenum cofactor biosynthesis, represented in purple, blue, and green, respectively. Insertion sequences are shown in red. Green and blue boxes indicate the target genomic regions for generating Δ*ndhL* and VC mutants, respectively. An internal fragment of the *ndhL* gene and the genomic region overlapping loci 04174–04175, located outside the *nic* gene cluster, was targeted. Both (c) Δ*ndhL* and (d) VC mutant of NtRootA2 were generated via a single crossover-mediated homologous recombination. The corresponding target regions were cloned into the pCR4-TOPO vector, indicated in green and blue, respectively. Vector insertion into target regions was confirmed by PCR amplification. Primer sets were designed to amplify the regions flanking cloning site, which were not amplified in the insertion mutants due to disruption by vector insertion. Fig. S2. Conjugation experiment for the horizontal transmission of the *nic* gene cluster. The donor strain NtRootA2 carried a plasmid with a kanamycin resistance gene. A spontaneous rifampicin-resistant mutant of strain StoSoilB3, which lacks the *nic* gene cluster, served as the recipient. Black triangles indicate the primer sets targeting donor- and recipient-specific genomic regions and the *ndhL* gene. Donor (Kan^R^/Rif^S^) and recipient (Kan^S^/Rif^R^) strains were co-cultured on LB medium. Transconjugants (Kan^R^/Rif^R^) were selected on LB medium containing 100 μg/mL kanamycin and 100 μg/mL rifampicin. Transmission of the *nic* gene cluster was confirmed by PCR amplification and a nicotine degradation assay. Fig. S3. Photograph of gnotobiotic cultivation setup. Inoculation assays were performed in a closed gnotobiotic biotic cultivation system using polycarbonate plant boxes (φ87 × ~ 121.5 mm, Bio Medical Science Inc., Japan). To maintain aeration, cotton plugs were inserted into the top ventilation hole. Prior to inoculation assay, approximately 18 g of vermiculite was added to each plant box and sterilized by the autoclaving at 121℃ for 20 min. Fig. S4. The *nic* gene clusters of *Arthrobacter* strains used for comparative genome analysis. The three sub-clusters of the *nic* gene cluster (γ-N-methylaminobutyrate catabolism, nicotine degradation, and molybdenum cofactor biosynthesis) are shown in purple, blue, and green, respectively. Insertion sequences are shown in red. IS elements flanking the *nic* gene cluster are indicated by white-filled arrows. (b) GC content of the *nic* gene cluster and its surrounding genomic regions in strain NtRootA9. Genomic regions flanked by IS elements and the surrounding regions are indicated by light blue and white arrows, respectively. IS elements flanking the *nic* gene cluster are shown in red and are indicated by white-filled arrows. The figure was generated using SnapGene software (www.snapgene.com). Fig. S5. Phylogenic analysis of the *nic* gene cluster and topology comparison with the the species tree. (a) The phylogeny of *nic* gene cluster was inferred based on the concatenated alignment protein sequences of nine core *nic* genes. The phylogenic tree was constructed using Fasttree. Bootstrap values above 70% are indicated by circle at nodes and node size is proportional to the bootstrap value. Branch colors represent the latest classification of the genus *Arthrobacter.* (b) Tanglegram shows the phylogeny of *nic* gene cluster relative to the species tree. The branch color of species tree represents the latest classification of the genus *Arthrobacter. *Fig. S6. The area of the growth curve (AUC) for In vitro growth assay of NtRootA2 and its derivative mutants. AUC was calculated based on OD_600_ measures over 24 h. Data points were analyzed based on a one-way ANOVA with Dunnett’s post hoc test. n.s., not significant. Data are presented as mean ± standard deviation with individual data points (n = 4–5). Control: 0 μM nicotine; Low, 1 μM nicotine; Medium, 10 μM of nicotine; and High, 100 μM nicotine. Fig. S7. Nicotine degradation assay of SynCom members. UPLC analysis of culture supernatants from each bacteria strain grown in nicotine-supplemented PBS. Chromatograms were recorded at 264 nm using identical intensity scales. a.c., arbitrary units. Essentially identical results were obtained from two independent experiments. Fig. S8. Mean relative abundance of single strains in the SynComs. Means ± SE, boxplots, and individual datapoints are shown (n = 10). Data were obtained from two independent experiments. The two-way ANOVA result table is provided. Different letters indicate statistical differences based on a two-way ANOVA followed by HSD test (α = 0.05). SynCom: wildSC, ndhLSC, or conSC. Plant: tobacco genotype (Wild-type or *erf189*/*erf199*). S × P: interaction between SynCom and tobacco genotype. Different letters indicate statistical differences corresponding to two-way ANOVA followed by HSD test (α = 0.05). Fig. S9. In vitro growth assay of SynCom members in TY medium supplemented with nicotine. (a) Growth curves (OD_600_) of each SynCom member in TY medium supplemented with nicotine. Data are expressed as mean ± SD (n = 6). Control: 0 μM nicotine (b) The area of the growth curve (AUC) was calculated based on OD_600_ measures measurements over 30 h. Data points were analyzed based on a *t*-test between Control and Nicotine treatments. n.s., not significant. Data are presented as mean ± standard deviation with individual data points (n = 6). Essentially identical results were obtained from two independent experiments.

Supplementary Material 2.

## Data Availability

The data set supporting the results of this study is publicly available at the DDBJ (https://www.ddbj.nig.ac.jp) (PRJDB11299 and PRJDB18225). BioSample Accession for individual Arthrobacter genome sequences is listed in Dataset S2. All source data and R code used for statistical analysis and graphing are available from GitHub (https://github.com/t-shimasaki/Shimasaki_et_al_Tobacco-SynCom).
