# Supplementary material for: Horizontal acquisition of nicotine catabolism gene cluster enhances Arthrobacter fitness within tobacco root microbiota
Source: Microbiome. 2026 Jul 15;14:227. doi: 10.1186/s40168-026-02466-x (PMC13651117; doi:10.1186/s40168-026-02466-x)
Supplement: Supplementary file 1 [file 40168_2026_2466_MOESM1_ESM.pdf]

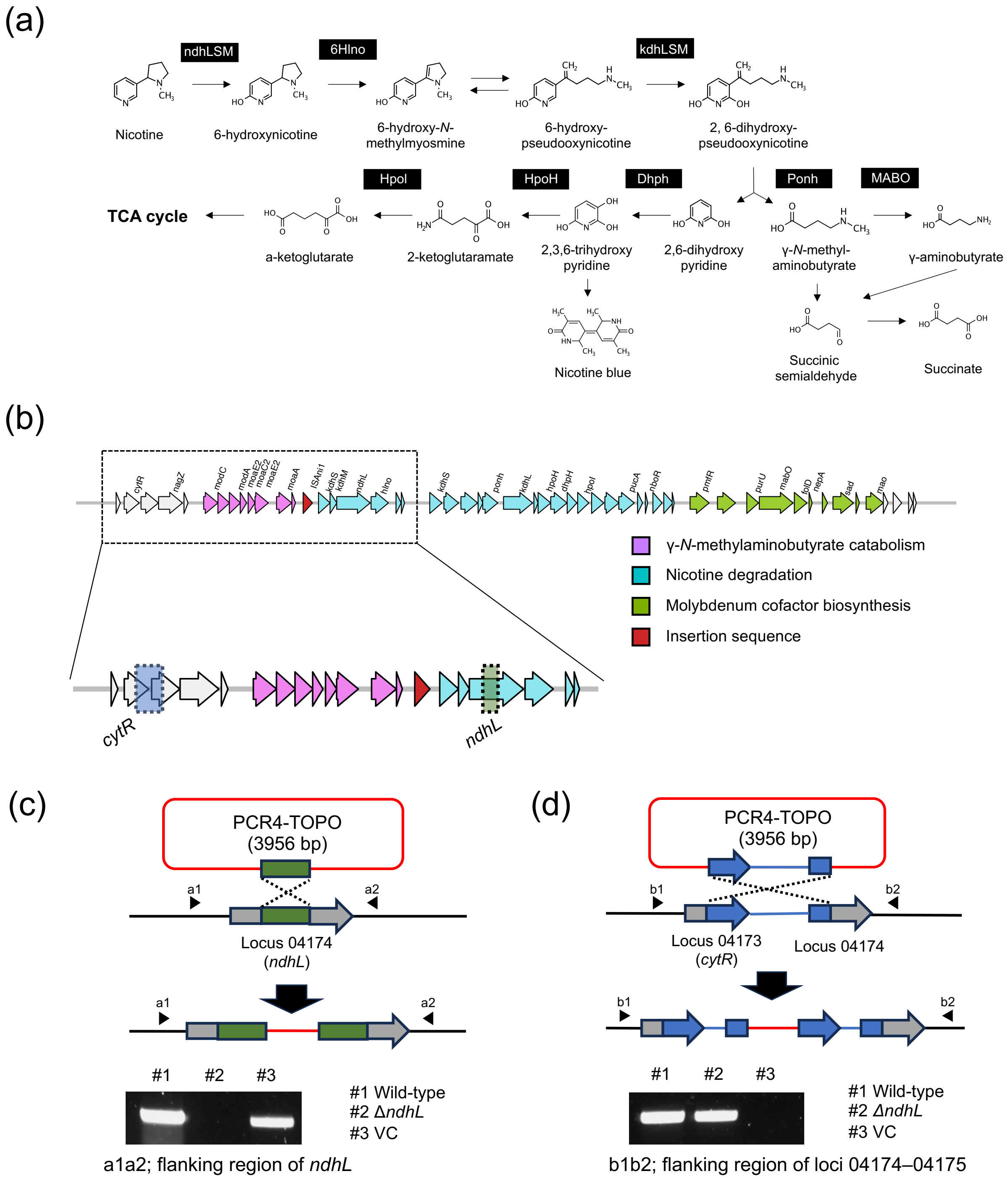

Fig. S1. Nicotine-catabolism pathway and *nic* gene cluster of *Arthrobacter* spp., and generation of NtRootA2 mutants. (a) Overview of the nicotine degradation by *Arthrobacter* spp. and **(b)** and *nic* gene cluster of NtRootA2. The cluster comprises three sub-gene clusters involved in γ-N-methylaminobutyrate catabolism, nicotine degradation, and molybdenum cofactor biosynthesis, represented in purple, blue, and green, respectively. Insertion sequences are shown in red. Green and blue boxes indicate the target genomic regions for generating Δ*ndhL* and VC mutants, respectively. An internal fragment of the *ndhL* gene and the genomic region overlapping loci 04174–04175, located outside the *nic* gene cluster, was targeted. Both **(c)** Δ*ndhL* and **(d)** VC mutant of NtRootA2 were generated via a single crossover-mediated homologues recombination. The corresponding target regions were cloned into the pCR4-TOPO vector, indicated in green and blue, respectively. Vector insertion into target regions was confirmed by PCR amplification. Primer sets were designed to amplify the regions flanking cloning site, which were not amplified in the insertion mutants due to disruption by vector insertion.

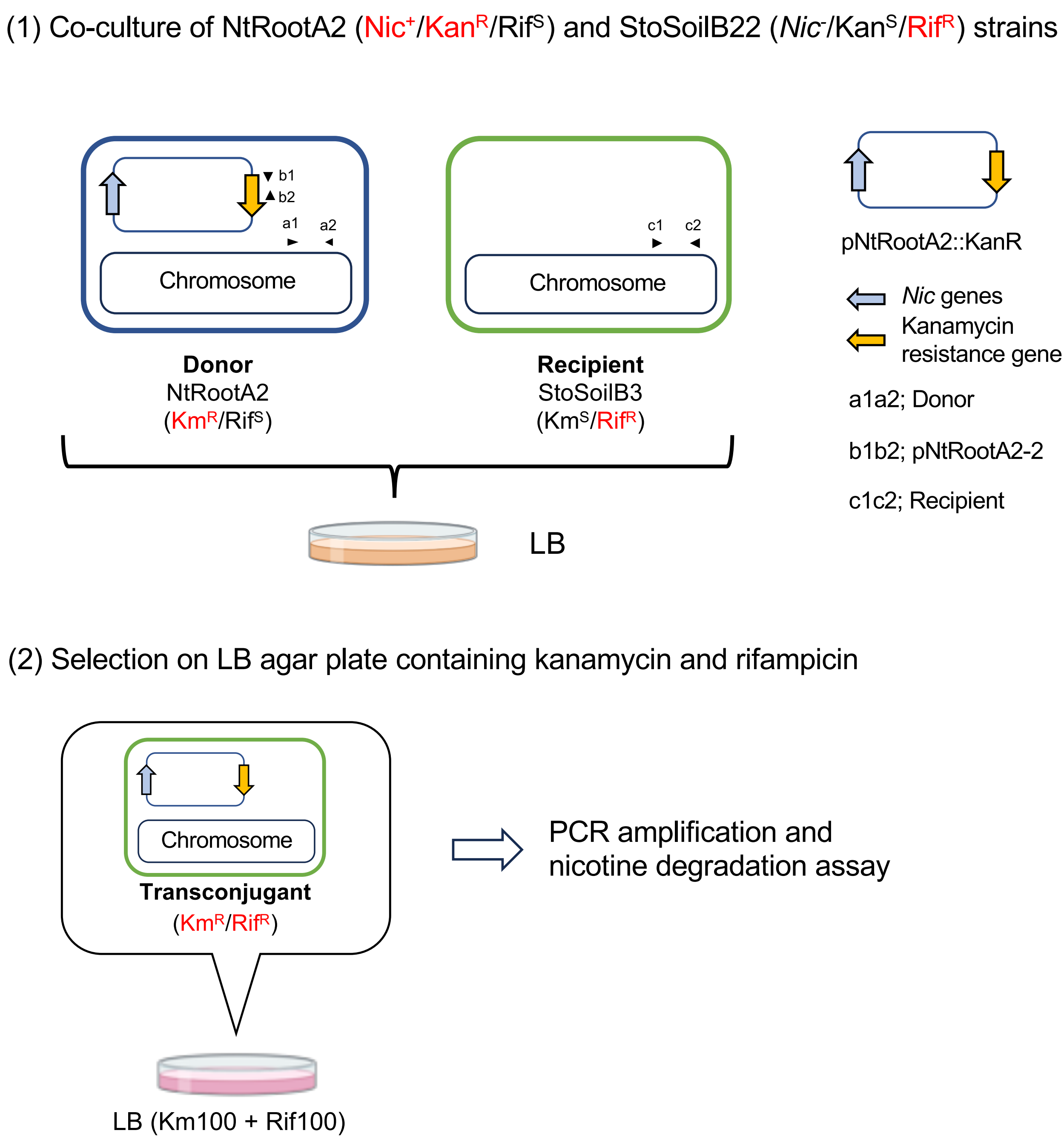

Fig. S2. Conjugation experiment for the horizontal transmission of the *nic* gene cluster. The donor strain NtRootA2 carried a plasmid with a kanamycin resistance gene. A spontaneous rifampicin-resistant mutant of strain StoSoilB3, which lacks the *nic* gene cluster, served as the recipient. Black triangles indicate the primer sets targeting doner- and recipient-specific genomic regions and the *ndhL* gene. Donor (Kan^R/Rif^S) and recipient (Kan^S/Rif^R) strains were co-cultured on LB medium. Transconjugants (Kan^R/Rif^R) were selected on LB medium containing 100 μg/mL kanamycin and 100 μg/mL rifampicin. Transmission of the *nic* gene cluster was confirmed by PCR amplification and a nicotine degradation assay.

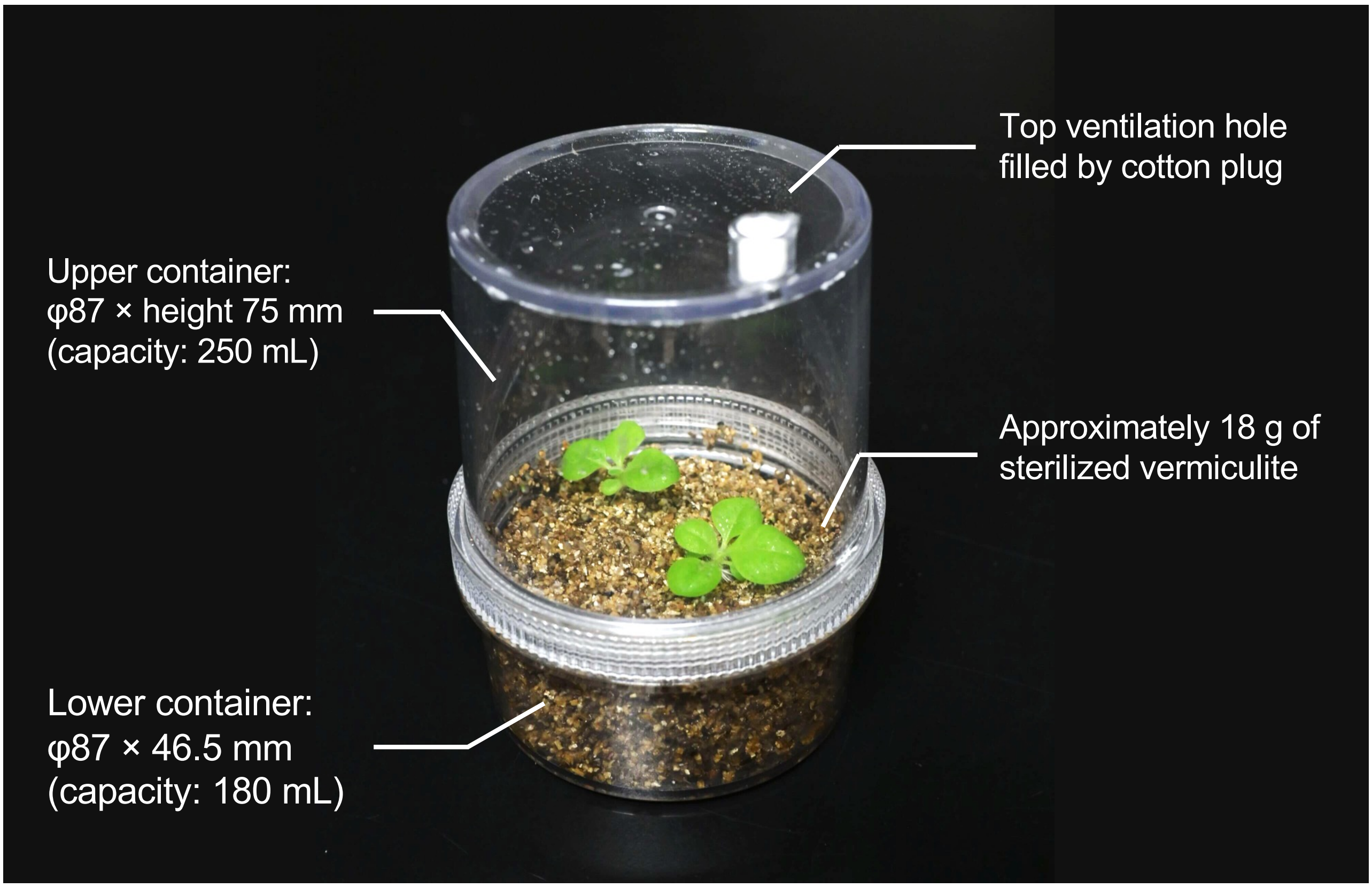

Fig. S3. Photograph of gnotobiotic biotic cultivation setup. Inoculation assays were performed in a closed gnotobiotic biotic cultivation system using polycarbonate plant boxes (φ87 × ~121.5 mm, Bio Medical Science Inc., Japan). To maintain aeration, cotton plugs were inserted into the top ventilation hole. Prior to inoculation assay, approximately 18 g of vermiculite was added to each plant box and sterilized by the autoclaving at 121°C for 20 min.

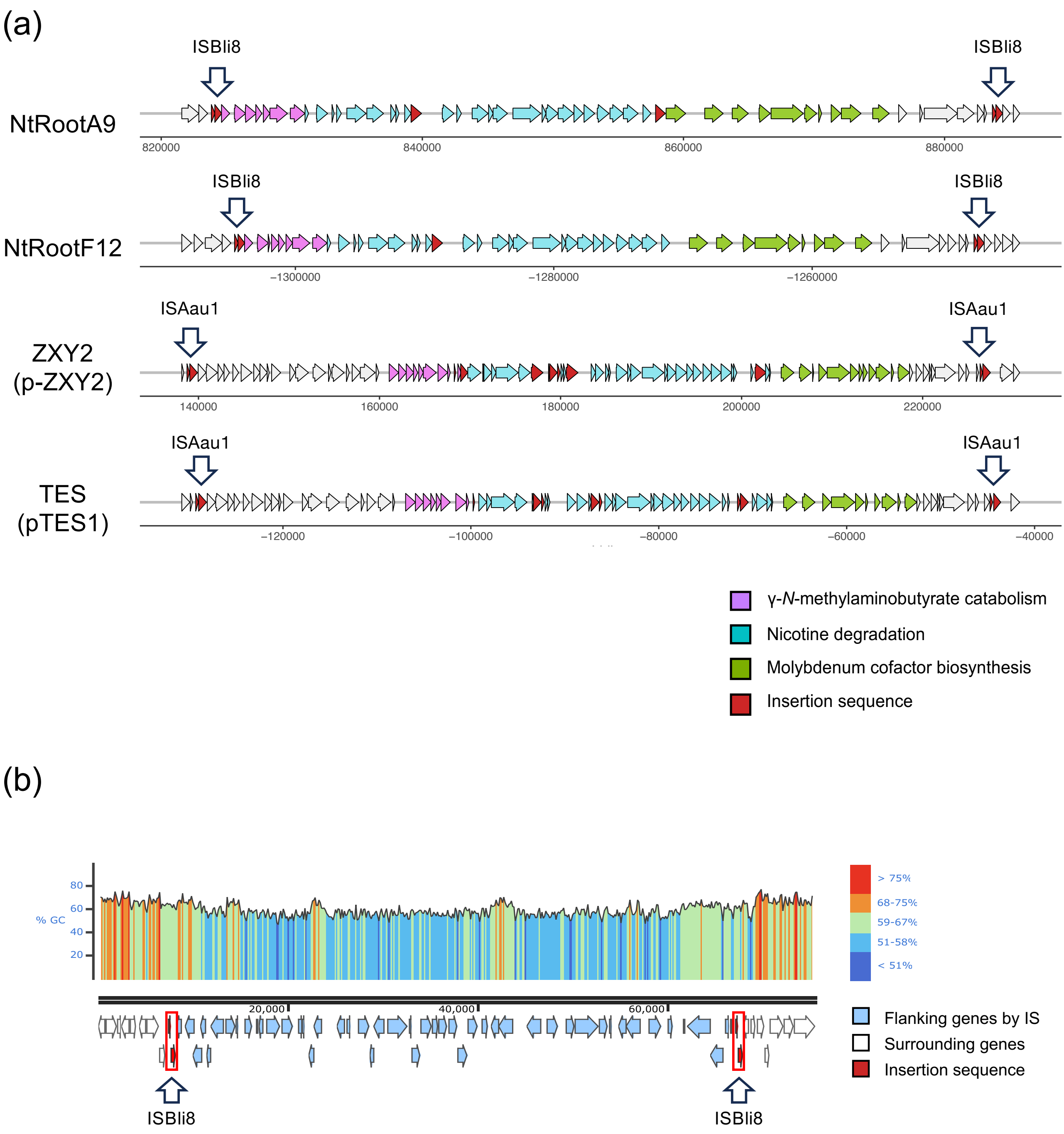

Fig. S4. The *nic* gene clusters of *Arthrobacter* strains used for comparative genome analysis. (a) The three sub-clusters of the *nic* gene cluster (γ-N-methylaminobutyrate catabolism, nicotine degradation, and molybdenum cofactor biosynthesis) are shown in purple, blue, and green, respectively. Insertion sequences are shown in red. IS elements flanking the *nic* gene cluster are indicated by white-filled arrows. **(b)** GC content of the *nic* gene cluster and its surrounding genomic regions in strain NtRootA9. Genomic regions flanked by IS elements and the surrounding regions are indicated by light blue and white arrows, respectively. IS elements flanking the *nic* gene cluster are shown in red and are indicated by white-filled arrows. The figure was generated using SnapGene software (www.snapgene.com).

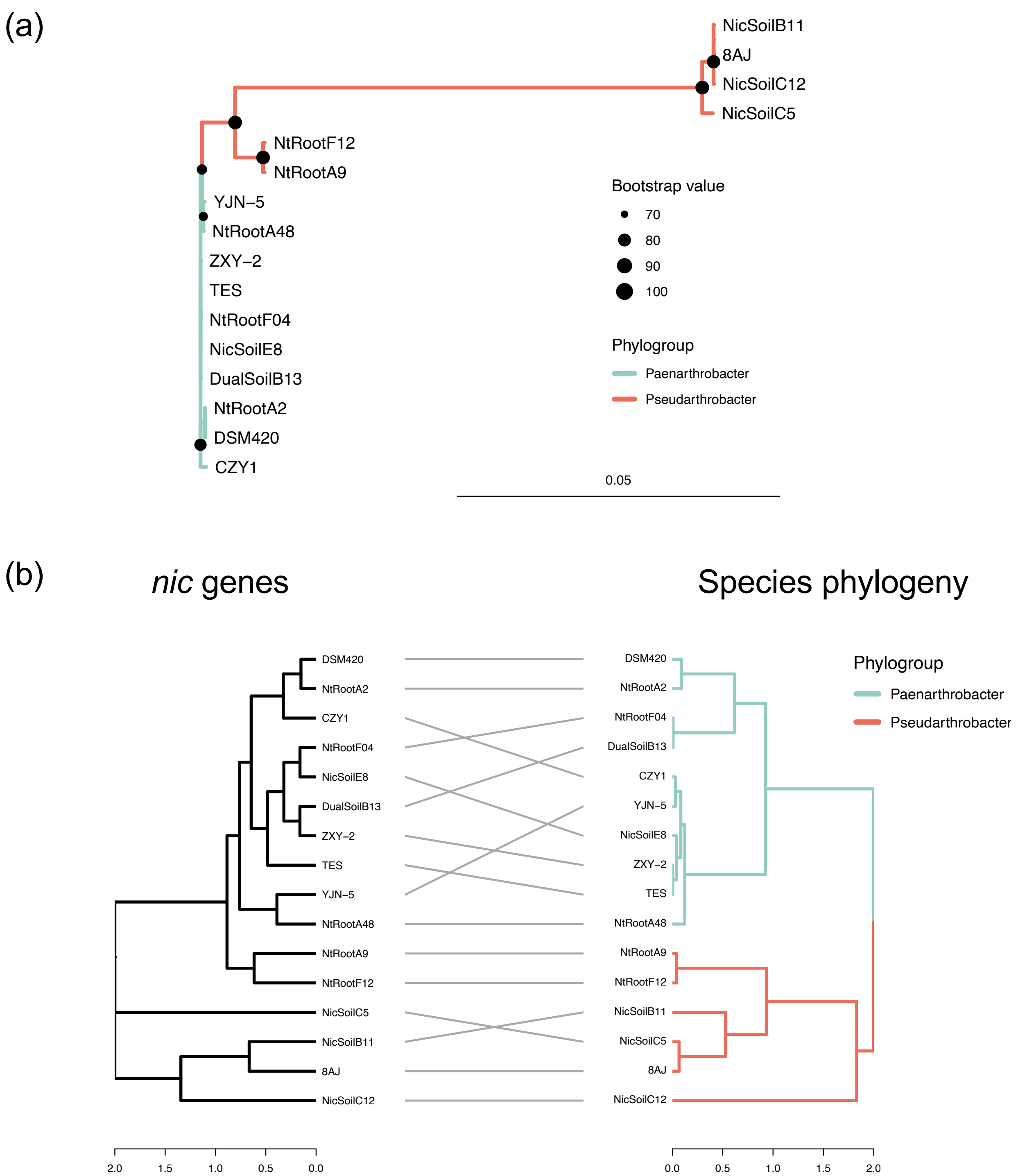

Fig. S5. Phylogenic analysis of the *nic* gene cluster and topology comparison with the the species tree. (a) The phylogeny of *nic* gene cluster was inferred based on the concatenated alignment protein sequences of nine core *nic* genes. The phylogenic tree was constructed using Fasttree. Bootstrap values above 70% are indicated by circle at nodes and node size is proportional to the bootstrap value. Branch colors represent the latest classification of the genus *Arthrobacter.* **(b)** Tanglegram shows the phylogeny of *nic* gene cluster relative to the species tree. The branch color of species tree represents the latest classification of the genus *Arthrobacter.*

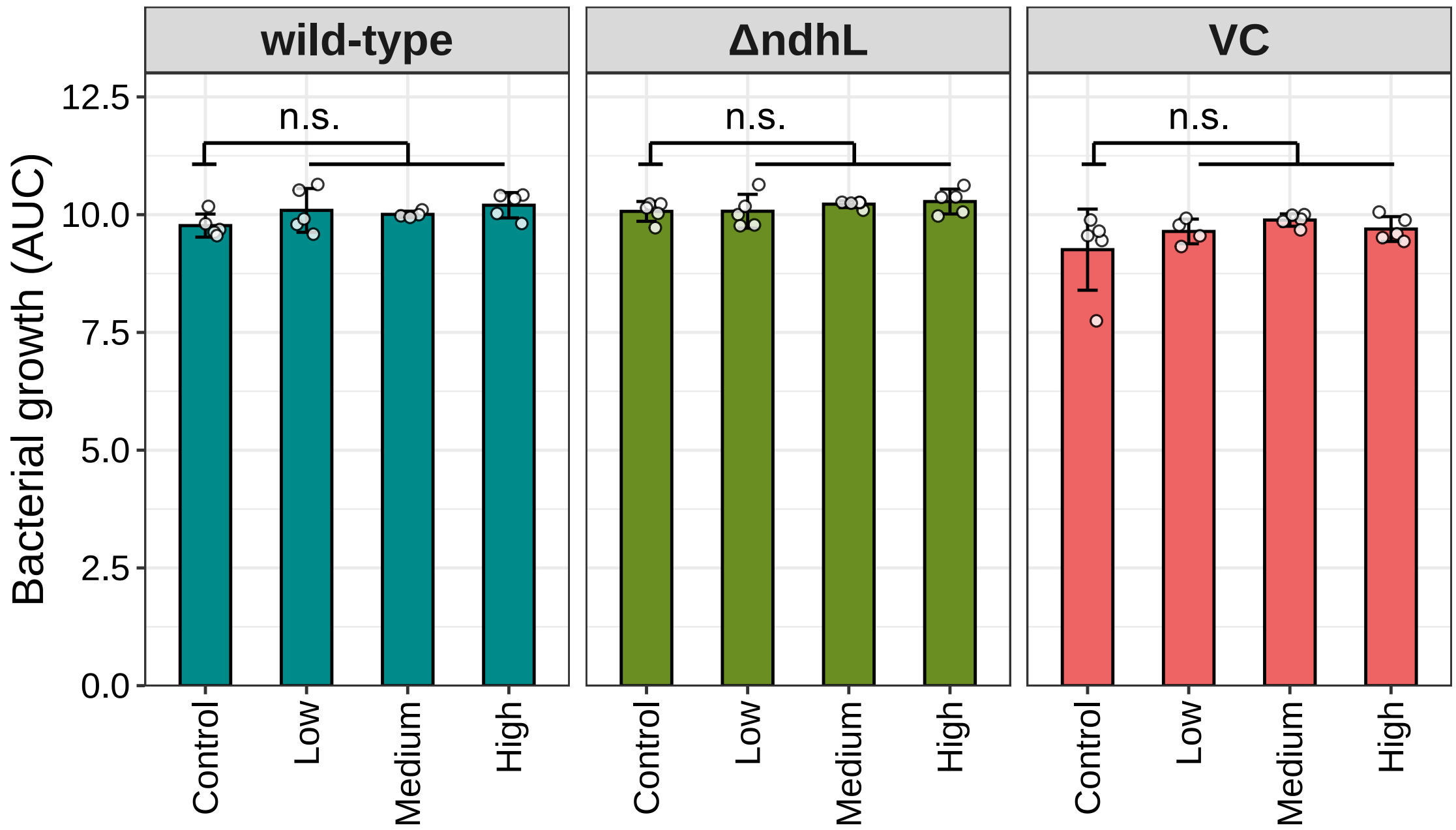

Fig. S6. The area of the growth curve (AUC) for *In vitro* growth assay of NtRootA2 and its derivative mutants. AUC was calculated based on OD_{600} measures over 24 h. Data points were analyzed based on a one-way ANOVA with Dunnett's post hoc test. n.s., not significant. Data are presented as mean ± standard deviation with individual data points (n = 4–5). Control: 0 μM nicotine; Low, 1 μM nicotine; Medium, 10 μM of nicotine; and High, 100 μM nicotine.

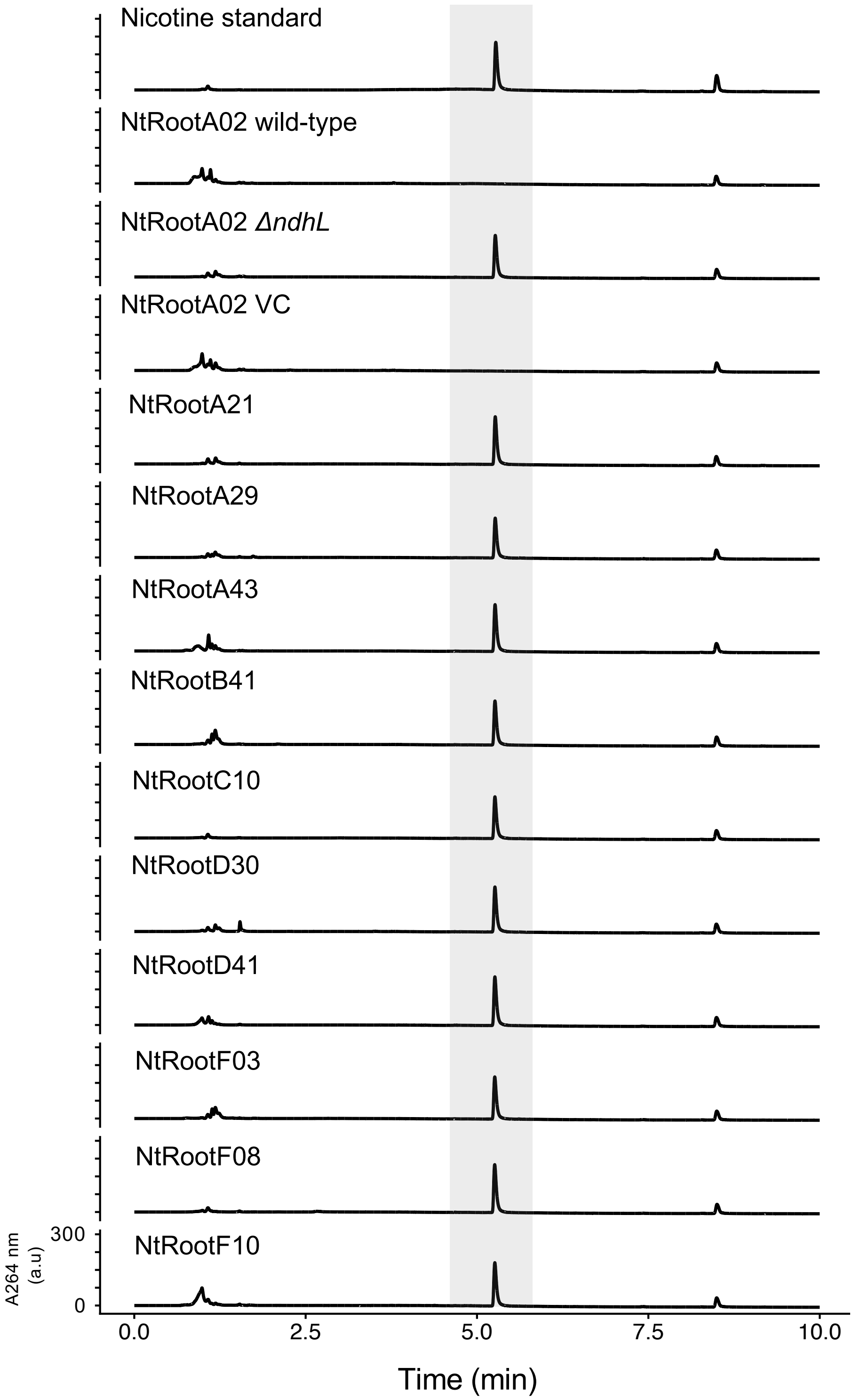

Fig. S7. Nicotine degradation assay of SynCom members. UPLC analysis of culture supernatants from each bacteria strain grown in nicotine-supplemented PBS. Chromatograms were recorded at 264 nm using identical intensity scales. a.c., arbitrary units. Essentially identical results were obtained from two independent experiments.

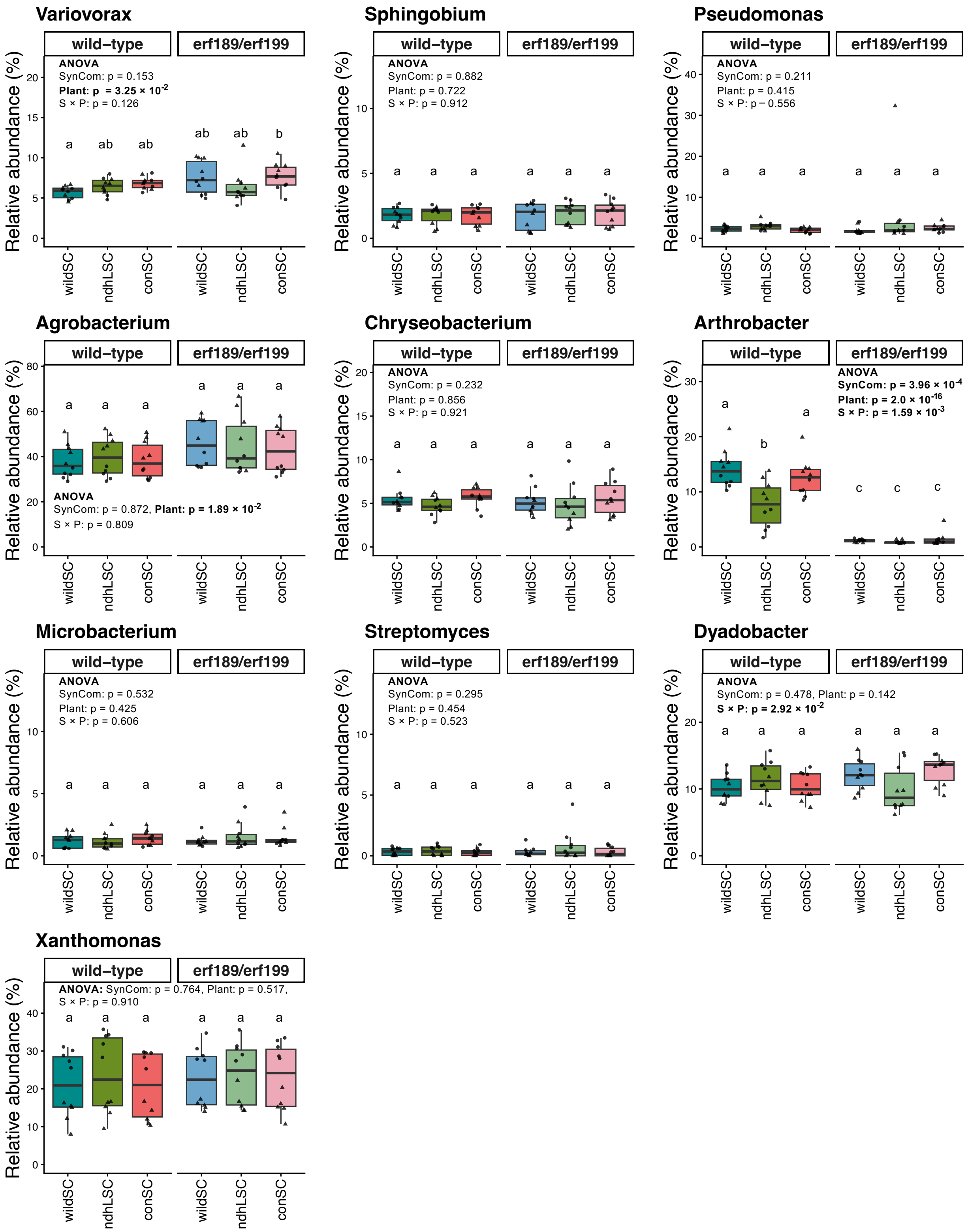

Fig. S8. Mean relative abundance of single strains in the SynComs. Means ± SE, boxplots, and individual datapoints are shown (n = 10). Data were obtained from two independent experiments. The two-way ANOVA results are reported inside of each figure. Different letters indicate statistical differences based on a two-way ANOVA followed by HSD test (α = 0.05). SynCom: wildSC, ndhLSC, or conSC. Plant: tobacco genotype (Wild-type or *erf189*/*erf199*). S × P: interaction between SynCom and tobacco genotype. Different letters indicate statistical differences corresponding to two-way ANOVA followed by HSD test (α = 0.05).

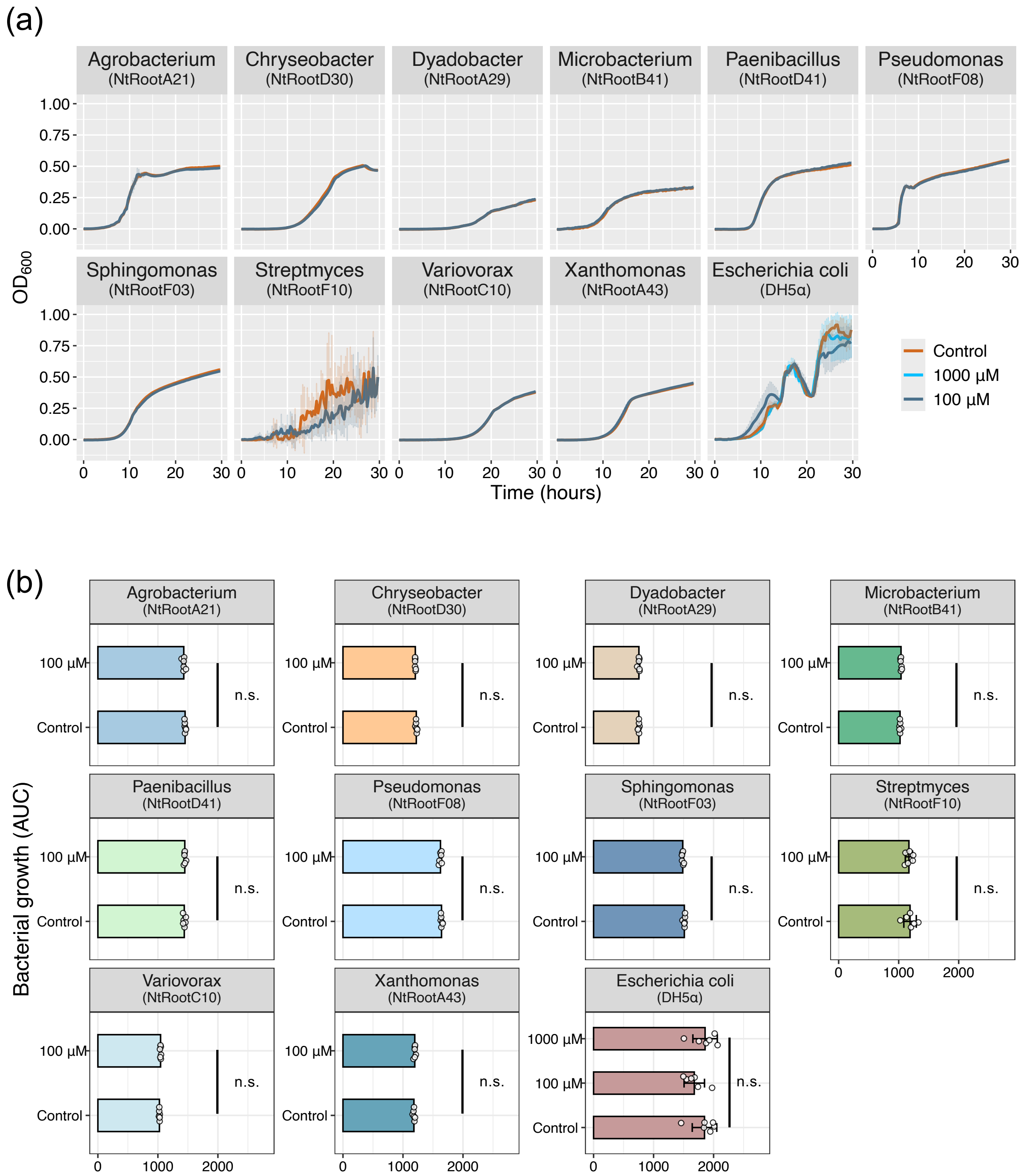

Fig. S9. *In vitro* growth assay of SynCom members in TY medium supplemented with nicotine. (a) Growth curves (OD_{600}) of each SynCom member in TY medium supplemented with nicotine. Data are expressed as mean ± SD (n = 6). Control: 0 µM nicotine **(b)** The area of the growth curve (AUC) was calculated based on OD_{600} measures measurements over 30 h. Data points were analyzed based on a *t*-test between Control and Nicotine treatments. n.s., not significant. Data are presented as mean ± standard deviation with individual data points (n = 6). Essentially identical results were obtained from two independent experiments.
